# Photonic crystal enhanced fluorescence using a hybrid hexagonal boron nitride spacer and plasmonic gold cryosoret cavity

**DOI:** 10.1039/d5nr02950c

**Published:** 2025-11-05

**Authors:** Souvik Bhattacharya, Seemesh Bhaskar, Weinan Liu, Joseph Tibbs, Vivek Pachchigar, R. Mohan Sankaran, Brian T. Cunningham

**Affiliations:** a Department of Nuclear, Plasma, and Radiological Engineering, The Grainger College of Engineering, University of Illinois, Urbana-Champaign Champaign IL USA rmohan@illinois.edu; b Materials Research Laboratory, University of Illinois Urbana-Champaign Urbana IL USA; c Department of Electrical and Computer Engineering, University of Illinois Urbana-Champaign Urbana IL 61801 USA bcunning@illinois.edu; d Nick Holonyak Jr Micro and Nanotechnology Laboratory, University of Illinois Urbana-Champaign Urbana IL 61801 USA; e Carl R. Woese Institute for Genomic Biology, University of Illinois Urbana-Champaign Urbana IL 61801 USA; f Department of Bioengineering, University of Illinois at Urbana-Champaign Urbana IL 61801 USA; g Department of Chemistry, University of Illinois Urbana-Champaign Urbana IL 61801 USA; h Cancer Center at Illinois Urbana IL 61801 USA

## Abstract

The introduction of photonic technologies in fluorescence-based detection platforms such as point-of-care diagnostics enables highly reliable quantitative and qualitative analysis. Photonic crystals (PCs) and plasmonic nanoparticles (NPs) have individually shown promise, and combining them with radiating dipoles is expected to yield synergistic effects. However, integration has been thus far hindered by severe fluorescence quenching as a result of metal–fluorophore proximity in the so-called ‘zone of inactivity’. Here, we show that ultrathin hexagonal boron nitride (hBN) can serve as an active insulating spacer to suppress nonradiative quenching while maintaining strong coupling between the localized surface plasmon resonance of gold nanoparticles and the guided mode resonance of an underlying photonic crystal. Furthermore, we fabricate tunable gold cryosoret nanoassemblies atop the fluorophore layer, creating nanocavity architectures that concentrate and amplify electromagnetic fields at the infinitesimal gap of radiating dipoles. This hybrid platform comprising photonic crystal-guided mode resonances, localized surface plasmon resonance, and Bragg–Mie hybrid modes from cryosoret assemblies is found to produce 650-fold enhancement, which corresponds to the attomolar limit of detection of a fluorescent reporter. Our study provides a new design strategy that maximizes fluorescence output while preventing detrimental energy loss pathways, a critical step for future applications in ultrasensitive biomarker detection and related technologies.

## Abstract 2

Electric and magnetic field hotspots enhanced using a hBN spacer with Au cryosoret nanocavities. The radiating dipole at the cryosoret–hBN–photonic crystal interface drives unprecedented photonic crystal enhanced fluorescence (PCEF).

## Graphical abstract

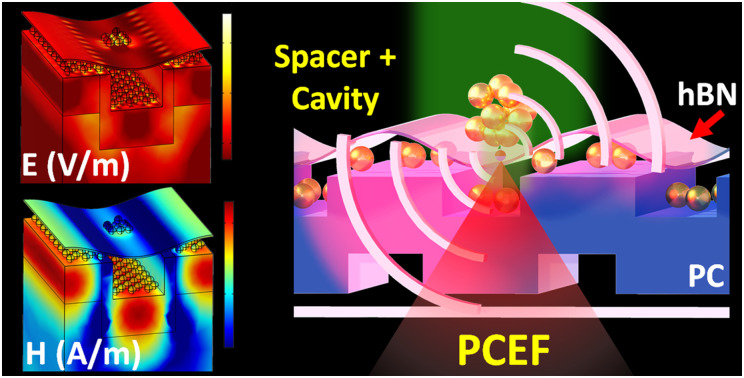

Photonic crystals (PCs) have gained tremendous attention in recent years owing to their high sensitivity, speed of response, and potential for miniaturization of the associated microscopy and spectroscopy modalities for trace level detection of biomarkers such as DNA, proteins, microRNAs, and even entire viruses.^1–3^ Combining all-dielectric lossless PC substrates presenting high-quality (high-Q) resonances with plasmonic nanoparticles (NPs), such as Ag, Au, Pt, Pd to name a few, offers additional possibilities to efficiently couple electromagnetic energy between the macro, micro, and nanoscale domains.^4,5^ Such hybrid systems provide fascinating experimental grounds for uncovering new physical phenomena, such as Fabry–Pérot mode coupling, fano resonances, hybridized plasmon–phonon polaritons (ferroplasmons), and manifestations of quantum coherence at nanogaps.^6–8^ Though the interactions between plasmonic NPs and fluorophores are typically complex, optimizing the experimental conditions can enhance local excitation fields, increase the radiative decay rates, and reduce lifetimes, which leads to fluorescence enhancement, a crucial aspect to augment the performance of associated biosensing devices.^9,10^

A major challenge for combining PCs and plasmonic NPs is the ‘quenching’ phenomena on account of the high extinction coefficients of noble metals at UV-Vis-NIR wavelengths.^11–15^ Additionally, there are proximity-induced quenching processes driven by Förster resonance energy transfer (FRET) and nanomaterial surface energy transfer (NSET) mechanisms (scaling inversely with the sixth and fourth powers, respectively) in the so called ‘zone of inactivity’, in which there is a separation distance of less than 5 nm between the metal and fluorophore.^16,17^ Such quenching effects have profound implications for biosensor applications as the signal-to-noise ratios are dramatically reduced with emergence of non-radiative decay channels.^18^ To circumvent these limitations and achieve fluorescence lifetime engineering (of molecules, quantum dots and nanodiamonds), our group previously developed photonic crystal enhanced fluorescence (PCEF) technology.^19–22^ PCEF leverages guided mode resonance (GMR) of the PC to amplify emission intensity without modifying the quenching pathways inherent in metals (both thin films and NPs).^23,24^ Using PCEF, we have demonstrated ultrasensitive detection of diverse analytes, including microRNAs, viral particles, proteins, DNA fragments, and exosomes.^25–29^ In principle, a biosensing strategy could be to utilize the broad spectral resonances (low-Q) of plasmonic NPs, which show small mode volume (strong Purcell enhancements), to generate photo-plasmonic hybrid ‘hotspots’.^30–32^ However, in scenarios where there is an effective overlap between the extinction of the plasmonic NPs and the radiating dipoles, pronounced fluorescence quenching has been observed that overwhelms the high field intensity of the PC substrate.^33,34^

In the past decade, several strategies have been investigated to overcome quenching by mitigating non-radiative losses while preserving or enhancing plasmonic benefits. A general approach that is suitable for sensor substrate development involves insertion of an insulating spacer layer between the fluorophore and metallic nanostructure coatings.^35^ Different materials including polymer films,^36^ inorganic ceramics,^37^ and two-dimensional (2D) materials such as graphene have been explored in this direction.^38,39^ Traditional polymeric spacers often require relatively thick layers (∼20–40 nm) to suppress quenching effectively, and their mechanical and thermal stabilities are suboptimal for multiple and long-term use.^35^ Inorganic oxide layers offer better thickness control and thermal endurance, but can contain defects that can cause undesirable charge tunnelling that lessen the fluorescence efficacy.^37^ Low-dimensional materials such as graphene provide improved mechanical and oxidative stability, but cannot avoid quenching because of their ability to accept excited-state electrons from the fluorophore *via* nonradiative energy transfer processes.^40^

Hexagonal boron nitride (hBN) is a van der Waals material that has attracted interest as a nanophotonic platform due to its ability to exhibit a variety of phenomena such as single-photon emission,^41–44^ second-harmonic generation^45^ and mid-IR hyperbolic phonon-polaritons.^46^ In particular, the emission properties of hBN have been widely exploited in diverse photonic applications, including single-photon sources, cavity-enhanced emission, and hybrid plasmonic architectures, underscoring its versatility in quantum and classical photonics.^42–44,47^ Very recently, atomically thin hBN has emerged as an ideal dielectric spacer material for plasmon-enhanced fluorescence (PEF) and surface-enhanced Raman scattering (SERS) applications.^35,40^ Though hBN is a layered and flexible structure that is mechanically similar to graphene, it is electrically insulating (unlike graphene).^48^ Consequently, it effectively blocks charge transfer pathways that lead to fluorescence quenching, in addition to demonstrating remarkable thermal stability, and structural integrity up to ∼800 °C in air,^35^ in stark contrast to graphene which oxidizes at approximately 300 °C. The van der Waals structure of hBN also allows it to accommodate very large strain gradients that preserve the integrity of the material even when placed on surfaces containing nanoparticles or patterned roughness. Moreover, hBN is characterized by exceptional impermeability (to gases and liquids), defect-free property at atomic scale, and good affinity for fluorophores (*via* π–π interactions).^40^ hBN has been found to enhance near-field optical effects responsible for electromagnetic hotspots and giant anisotropy, earning the designation of ‘record breaking material’.^49^

In this report, we present integration of insulating high refractive index (HRI) hBN with PC substrates that are pre-decorated with plasmonic AuNPs to suppress fluorescence quenching by simultaneously leveraging the high-Q GMR of the PC and low-mode volume LSPR of AuNPs. To maximize the fluorescence output, we further incorporated gold cryosoret (AuCS) nano-assemblies^20,21,50,51^ on top of the PC–AuNP–hBN–dye substrate. Consequently, optical nanocavities with infinitesimal nano-gaps are generated that trap and recycle emitted photons from the radiating dipoles sandwiched between the PC–AuNP–hBN from below and AuCS from above.^52,53^ This architecture is found to generate strong metallo-dielectric hotspots, leading to 650-fold fluorescence enhancement.^53,54^ The experimental measurements are supported by COMSOL, RWCA and FEM simulations that visualize the presence of an unprecedented three-way photonic–plasmonic coupling between the PC modes, the LSPR of AuNPS, and the resonances of AuCS nano-assemblies, with spatial confinement orders of magnitude higher than their individual counterparts. By combining cryosoret nanoengineering with an hBN-mediated dequenching approach, this report establishes a versatile and scalable route to realize ultra-bright fluorescence emission for molecular sensing.^55–57^

In order to obtain large-area, continuous films that could be transferred onto a PC to serve as a dielectric spacer, hBN was grown by chemical vapor deposition (CVD), as schematically illustrated in Fig. 1a (see Methods for details). Briefly, ammonia borane (Boron Specialties, 99%) was sublimed and carried by a gas flow containing a mixture of argon and molecular hydrogen into a furnace and deposited at low pressure onto copper substrates. The optical microscopy image in Fig. 1b shows that the grown films are continuous over several millimeters and up to centimeters in size (see inset of Fig. 1b).

**Fig. 1:**
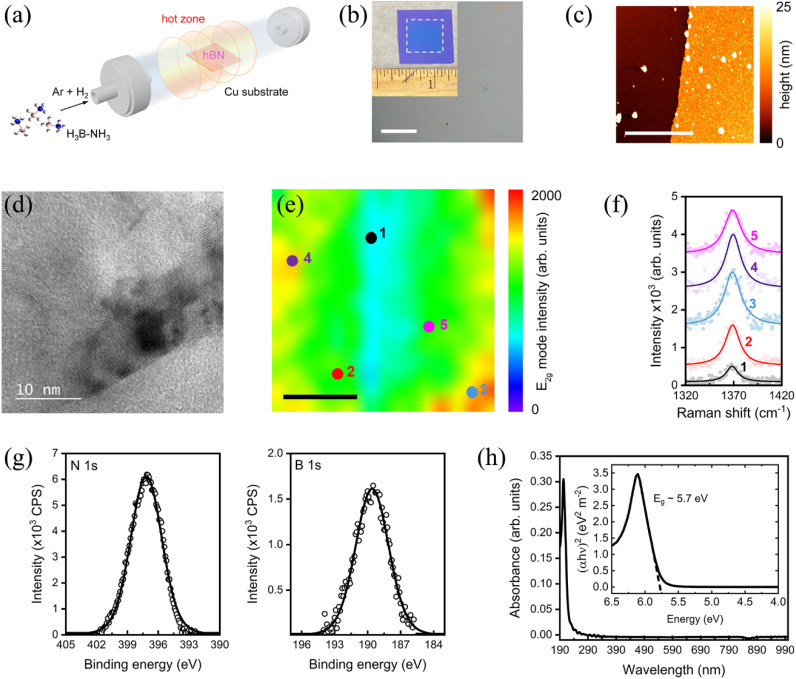
Synthesis and characterization of large-area hBN films. (a) Schematic illustration of the experimental setup used to grow hBN thin films on copper foils using ammonia borane as the molecular precursor. (b) Optical microscopy image of hBN film transferred to SiO_2_/Si substrates. The scale bar corresponds to 200 μm. Inset shows a photograph of a centimeter-scale film after substrate transfer. (c) AFM topography scan of the hBN film edge. The lateral scale bar corresponds to 5 μm. (d) HRTEM image of a folded edge of the hBN film showing lattice fringes indicating a crystalline structure. (e) Raman intensity map corresponding to the E_2g_ phonon mode (*ca.* 1370 cm^−1^) of hBN film. The scale bar corresponds to 3 μm. (f) Raman spectra collected from different spots of the hBN film in (e) showing the E_2g_ mode. (g) XPS of hBN film showing N 1s and B 1s regions near 397 eV and 190 eV, respectively. (h) UV-vis absorption spectra of hBN film showing a singular strong absorption in the UV at *ca.* 200 nm. Inset shows the derived Tauc plot which provides the optical bandgap estimated to be ∼5.7 eV.

To transfer hBN onto the photonic crystal substrates polymethyl methacrylate (PMMA)-assisted wet transfer method was utilized due to its well-established advantages for 2D material handling. PMMA can be spin-coated to precise thicknesses, is compatible with low-temperature processing (∼80 °C) necessary for oxidation-sensitive materials, and is insoluble in water, making it ideal for use with aqueous etchants such as ammonium persulfate for Cu removal. After etching, the PMMA/hBN stack can be thoroughly rinsed in DI water to remove residual etchants, ensuring a clean interface on the target substrate. PMMA can subsequently be removed using acetone or thermal treatment, leaving a pristine hBN surface. The adhesion baking temperature was determined empirically for the photonic crystal substrate, to be ∼110 °C, slightly higher than typical SiO_2_/Si transfers (∼90 °C), likely due to differences in surface functionality. While other polymers could potentially be used for wet transfer, PMMA provides an optimal combination of processability, chemical compatibility, and clean removal, supporting reproducible and high-quality hBN integration in our experiments.

To assess the thickness of the grown films, atomic force microscopy (AFM) topography imaging was conducted as shown in Fig. 1c. The representative hBN film is measured at the edge to be ∼3–4 nm thick with a RMS roughness of ∼1.5 nm. The crystalline structure of the film was confirmed by high-resolution transmission electron microscopy (HRTEM) imaging, which shows well-defined lattice fringes (Fig. 1d), and Raman mapping, which shows the E_2g_ mode at *ca.* 1370 cm^−1^ throughout a 10 × 10 sq. µm area with minimal variations in intensity (Fig. 1e and f). The FWHM of the mode varied between 17–21 cm^−1^, which is on par with or better than commercial hBN films.^58^

Specifically, we analyzed the domain size of our hBN films using Raman spectroscopy, where the FWHM of the E2 g Raman mode (17–21 cm^−1^) corresponds to a domain size of ∼10 nm. Because the laser spot size used in our fluorescence measurements (∼1 mm) is several orders of magnitude larger than the polycrystalline grain size, the measured enhancement represents an average over many grains and grain boundaries. We also considered morphological variations introduced by wet transfer, such as micron-scale wrinkles and angstrom-scale point defects. These were similarly averaged out under our large illumination area, and control fluorescence measurements on as-transferred hBN showed negligible background signal under our probe conditions. Together, these results suggest that grain boundaries and defects have minimal impact on the uniformity and reproducibility of the observed fluorescence enhancement.

X-ray photoelectron spectroscopy (XPS) shown in Fig. 1g reveal N 1s and B 1s peaks at *ca.* 397 eV and 190 eV, respectively, with an areal ratio of 1.05, which indicates stoichiometric hBN and corresponds well with previous reports of sp^2^-hybridized hBN films.^59^ Finally, a representative ultraviolet-visible (UV-Vis) absorption spectrum shown in Fig. 1h speaks to the transparency of the film in the visible range. From a Tauc plot, the optical bandgap is estimated to be *ca.* 5.7 eV (see inset of Fig. 1h), which is close to the ideal theoretical value of hBN.^60^ We note that these properties, in particular the optical transparency, is critical for integration with PCs as a dielectric spacer with minimum absorption losses in the wavelength range where fluorescence coupling is desired.

After successful synthesis of large-area, continuous hBN films, the same wet transfer process detailed previously was used to transfer the films onto PC substrates in order to create heterostructures, as schematically shown Fig. 2a. The PC is a TiO_2_ coated glass substrate patterned in a linear grating structure which has a period of ∼380 nm, within which the ∼190 nm grooves are ∼100 nm deep. hBN films were transferred onto bare PCs and PCs coated with 20 nm diameter AuNPs (see Methods for details). Fig. 2b–e show AFM and SEM images at low and high resolutions of one edge of a representative hBN film on bare PC. Fig. 2f–i show similar low and high magnification AFM and SEM images of a representative heterostructure consisting of hBN and Au-coated PC (PC + Au + hBN). In both cases, hBN is found to cover the patterned substrate smoothly and uniformly, highlighting the presence of a well-defined interface. Furthermore, the film is observed to be continuous over several microns on both substrates indicating minimal transfer-induced structural damage to the hBN. Notably, AFM images show that the film follows a wave-like topography induced by the patterned grating of the underlying PC, but at the same time, is not fully conformal with the surface of the PC. Such structures demonstrating gaps at the interface have been a topic of interest in the broad domain of photo-plasmonics to concentrate field intensity to micro-nano-dimensions. Our interfacial engineering of hBN and PC with AuNPs in between them demonstrates a relatively straightforward wet-transfer procedure that is viable for using hBN and analogous low-dimensional materials in PCEF-based applications.

**Fig. 2:**
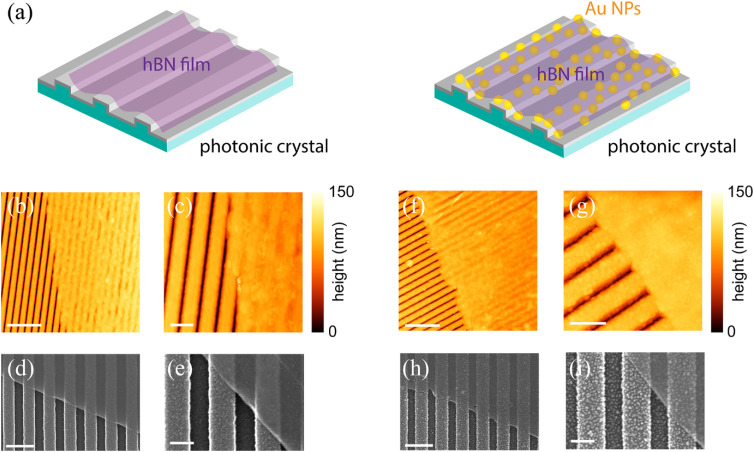
Interfacial engineering of hBN/PC and hBN/Au-NP/PC heterostructures. (a) Schematic illustration of the fabricated heterostructures comprising hBN films stacked on a bare PC (left) and an Au-nanoparticle decorated PC (right). (b) Low and (c) high resolution AFM topography images showing the edge of a representative hBN film on bare PC. (d) Low and (e) high magnification SEM images of the film edge for the samples shown in (b) and (c). (f) Low and (g) high resolution AFM topography images showing the edge of a representative hBN film on Au-NP/PC. (h) Low and (i) high magnification SEM images of the film edge for the samples shown in (f) and (g). The AFM color bars represent the height of features in the vertical direction. Scale bars for (b) and (f) correspond to 2 μm and (c) and (g) correspond to 0.5 μm. Scale bars for the SEM images in (d) and (h) correspond to 500 nm, and (e) and (i) correspond to 200 nm.

Fig. 3a shows the optical setup consisting of a light source (LS), collimating lenses (L1, L2), polarizer (P), and optical mount stage used for collection of angle-dependent transmittance spectra. Additional details of the setup and physics principles involved in the generation of the dispersion diagrams have been previously reported.^20,21,61^ The conceptual schematic of the generation of a spacer layer over the PC substrate is shown in Fig. 3b where the hBN nanolayer is placed over the PC as well as a PC + AuNP substrate. Prior to engineering the substrate (in our case, the PC) with different nanomaterials, it is vital to experimentally verify the optical response of the PC using dispersion diagrams (that are obtained theoretically). Dispersion diagrams can be used to delineate the fundamental properties of Bloch surface waves,^11,62,63^ internal optical modes,^64,65^ band edge modes^20^ and GMR effects.^21,61^Fig. 3c and f present the simulated dispersion diagram for TM and TE modes and the respective values are extracted and represented as yellow stars in Fig. 3d and g where the shaded data is the experimentally-obtained transmittance data. Further, to yield better understanding of the optical response of the PC under study, we present the experimental transmittance data for TM and TE polarizations in Fig. 3e and h, respectively. The analysis shows an excellent correlation between the simulated and experimental transmittance of the PC, underscoring the high structural robustness and quality of the substrate.

**Fig. 3:**
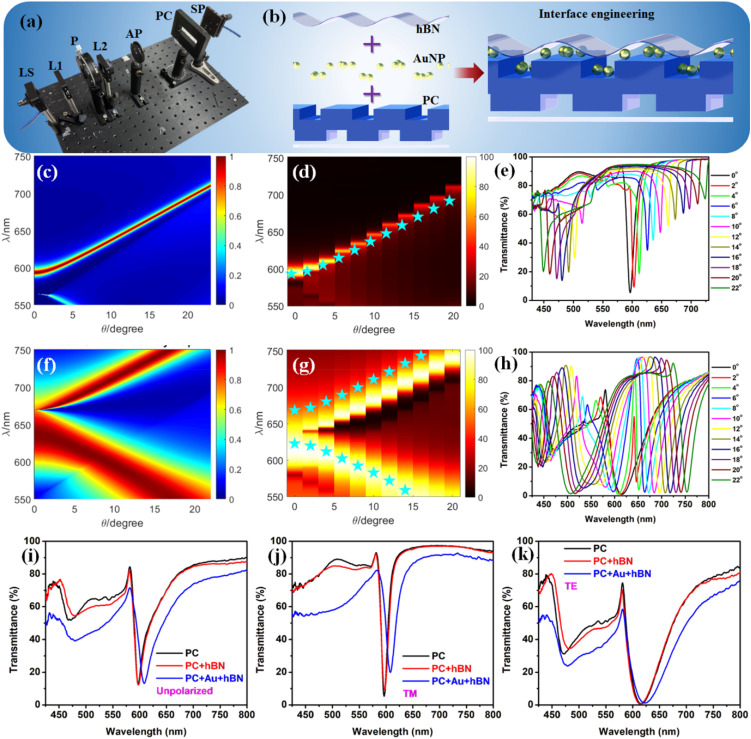
Photonic crystal optical response with hBN and AuNPs. (a) Photo of transmittance measurements setup (LS = light source; L1, L2 = lenses; P = polarizer; AP = aperture; PC = photonic crystal; SP = spectrometer). (b) Conceptual schematic of the generation of the spacer nanolayer over the PC substrate using AuNPs and hBN film. TM dispersion diagram obtained using (c) simulations and (d) its overlap with the experimentally-measured transmittance (shaded region) with the stars indicating the simulated data. (e) Wavelength *vs.* %*T* for TM optical response of the PC. TE dispersion diagram obtained using (f) simulations and (g) its overlap with the experimental transmittance (shaded region) with the stars indicating the simulated data. (h) Wavelength *vs.* %*T* for TE optical response of the PC. %*T vs.* wavelength experimental data obtained for PC, PC + hBN, and PC + Au + hBN systems for (i) unpolarized, (j) TM polarization, and (k) TE polarization.

While introducing a spacer nanolayer between AuNPs and the subsequently coated dye molecules reduces quenching, it is important to note that the addition of plasmonic AuNPs or hBN must not compromise the resonance of the PC itself. To understand this aspect, we have performed transmittance measurements of the PC, PC + hBN, and PC + Au + hBN multistacks using different polarizations as shown in Fig. 3i, j and k. A slight red-shift in the transmittance resonance dip of the PC with the addition of hBN and AuNPs is due to the change in the local dielectric constant at the PC interface. Nonetheless, the resonance remains stable (did not decrease/increase in intensity) regardless of coating hBN and AuNPs. This is on account of the small sized AuNPs and very thin hBN film (AFM and SEM images shown in Fig. 2) in building the multistack system. We expect that a thicker hBN film or larger sized AuNPs (or multilayers of AuNPs) would result in substantially deteriorating the strong optical response of the multistack system. Hence, the two crucial aspects for designing the multistack are as follows: (i) the hBN film thickness and AuNPs size should be kept small so as to retain a stable optical resonance of the underlying PC and (ii) the larger sized AuNPs and thicker hBN films should not effectively couple with the GMR of the PC as structures/multistack bigger than ∼200 nm would not effectively couple with the PC's evanescent field generated at resonance.

For applications in fluorescence-based biosensing, a major goal has been to augment the global fluorescence enhancements observed at the detector.^66,67^ While introducing a spacer hBN between the radiating dipoles and the underlying AuNPs on PC yields pathways to avoid quenching, it is quintessential to utilize hybrid “spacer + cavity” interfaces to observe not only dequenched, but also amplified fluorescence. Here, we synthesized cryosoret nano-assemblies as shown schematically in Fig. 4a by cooling pristine AuNPs in liquid nitrogen (−196 °C) whereby adiabatic cooling overcomes electrostatic repulsion.^50,51,68–70^ Collective hybridization of localized Mie modes (associated with individual NPs) and delocalized Bragg modes (emerging from nano-assemblies) results in new resonance features with enhanced field confinement and spectral tunability.^32,51,71^ The conceptual schematic of the generation of hybrid “spacer + cavity” interface using the synthesized cryosorets is shown in Fig. 4b. Fig. 4c–g present TEM images of the Au cryosorets (AuCS) obtained by cooling the AuNPs for 15 seconds, 30 seconds, 1 min, 2 min, and 3 min, respectively, yielding crysorets with 2 (±1), 5 (±2), 7 (±2), 9 (±3), and 12 (±3) particles per assembly. HAADF images of CS1 and CS5 in Fig. 4h and i, respectively, confirms the formation of robust cryosorets where the stable nano-gaps between the AuNPs comprising the cryosorets are vivid without any agglomeration (that would result in precipitation due to higher mass). The fluorescence signal intensity spectra observed for different sample variants (PC, PC + AuNPs, PC + AuNPs + hBN, PC + AuNPs + hBN + AuCS) are shown in Fig. 4j. The experimentally calculated fluorescence enhancements are shown in Fig. 4k. The formula used for the enhancement calculation is the direct ratio of the variant fluorescence intensity counts to that of the intensity counts on glass (with all experiments performed under identical experimental conditions)^20,21,61^ and is given as follows:1
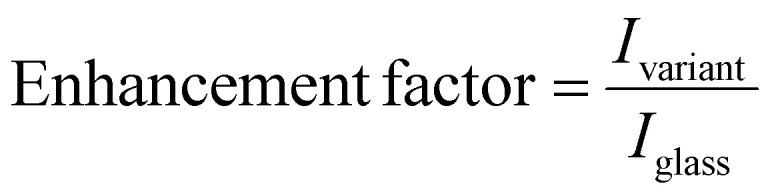


**Fig. 4:**
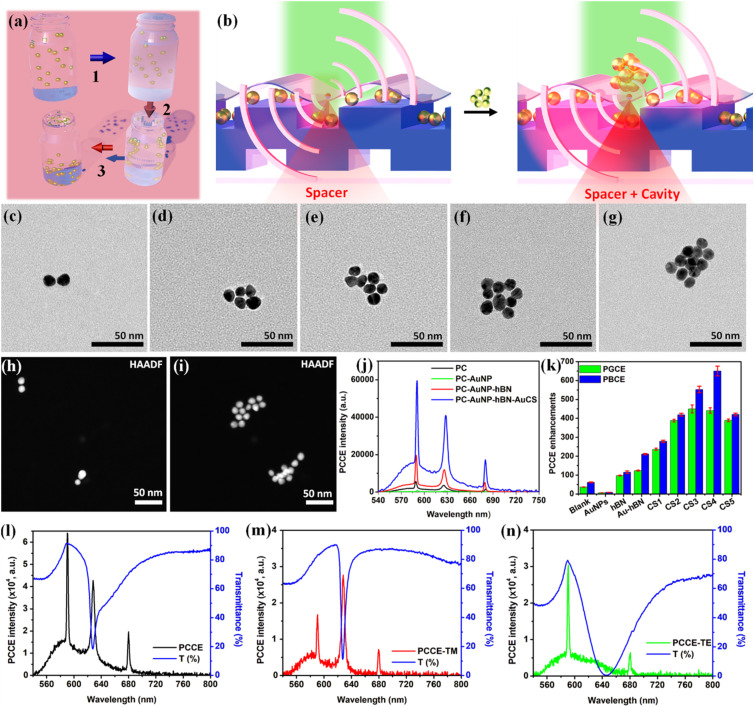
Photonic crystal enhanced fluorescence using hBN and Au crysoret nano-assemblies. (a) Illustration of the scheme for synthesis of cryosorets showing (1) adiabatic cooling, (2) thermos-migration and (3) separation of cryosorets. (b) Conceptual schematic of the generation of cavity nanointerface using plasmonic AuCSs over the PC pre-coated with AuNPs and hBN, where the hBN functions as a spacer. (c–g) TEM images of freshly prepared cryosorets (CS) nano-assemblies of gold named as CS1–5. (h and i) HAADF images of CS1 (dimer) and CS5 (multimer) shown as representative examples depicting the intact nanogaps rendered by the nano-assemblies. (j) Photonic crystal-coupled emission (PCCE) intensity for bare PC, PC with hBN, PC with AuNP + hBN, and PC with AuNP + hBN + AuCS. (k) PCCE enhancements obtained for different CSs and multistack configurations. Overlap of the experimental transmittance with the experimental fluorescence obtained for (l) unpolarized, (m) TM polarized and (n) TE polarized emission. The AuCS data shown here and in Fig. 4j is for CS4 which yielded the highest fluorescence enhancements (Fig. 4k).

Fig. 4k shows the enhancements for the PC band edge coupled emission (PBCE) and PC guided-mode coupled emission (PGCE), the occurrence and importance of which are elaborated in our recent report.^20^ Without any plasmonic nanomaterials, a bare PC yielded 60-fold enhancement in fluorescence as compared to free space. Quenching in the fluorescence intensity was observed with the addition of AuNPs (PC + AuNPs: 7-fold; Fig. 4j and k). In comparison, introducing the spacer hBN nanolayer resulted in an 115-fold enhancement for PC + hBN and 211-fold enhancement for PC + AuNPs + hBN. This doubling of the fluorescence enhancement can be attributed to the coupling between the resonances of the PC and LSPR of the AuNPs where hBN simultaneously offers pathways for quench-free radiative decay of emitters. Further interfacing CS1–CS5 yielded a steady increase from CS1 (280-fold) to CS4 (650-fold) before declining for CS5 (420-fold). This trend is in accordance with photo-plasmonic coupling of tunable nano-assemblies established in earlier works using plasmonic interfaces^20,21,61^ and fluorescence and interferometric scattering microscopy.^50^Fig. 4l–n indicate that the platform follows the radiating GMR model where the emitted photons carry the spectral feature as well as the polarization selectivity of the underlying PC to the far-field. Interfacing the cryosorets over the PC coated with AuNPs + hBN creates tunable multi-architecture nanogaps, where sandwiching radiating dipoles in the resultant cavity between the cryosorets and substrate generates EM hot spots with field enhancements of several orders of magnitude.^13,72–74^ These confined fields substantially enhance the excitation and emission intensity where the radiative decay rate increases with a concomitant decrease in lifetime (Fig. S6, SI). On account of its high chemical stability, large bandgap, and low-loss optical attributes, hBN serves as an ideal interlayer spacer for controlling radiative and non-radiative energy transfer processes. While the spacer layer avoids quenching effects, it's very thin profile still allows sufficient near-field overlap for field enhancement. While the pristine PC showed a 2.11 ns lifetime for the radiating dipoles, the addition of hBN resulted in a biexponential decay (1.05 ns, 64.1%; 2.72 ns, 35.9%). This behavior can be attributed to two different near-field effects experienced by the radiating dipoles (GMR of PC and HRI hBN). Similarly, a biexponential decay (1.68 ns, 65.9%; 2.77 ns, 34.1%) was observed for PC + AuNPs. Adding the hBN layer on top of the PC + AuNP hybrid resulted in a triexponential decay (1.00 ns, 59.2%; 2.32 ns, 11.9%; 2.90 ns, 28.9%), once again due to the combined influence of three components in determining the local field intensity. Further, incorporating the AuCSs on top of the PC + AuNPs + hBN system produced a significant reduction in lifetime (0.64 ns, 40.2%; 1.47 ns, 7.65%; 2.30 ns, 52.1%), indicating multiple radiative decay channels. These lifetime reductions directly correlate with the experimentally observed enhancement in PC-coupled fluorescence (details shown in SI).

Generating stable and reproducible plasmonic hotspots demands effective light harvesting where electric and magnetic flux densities at the nanoscale are harnessed. Our system utilizes AuCS as a highly tunable secondary material to form a coupled resonator with the bottom primary AuNP-PC substrate. The near-field E-field and H-field distribution plots for different multistack configurations are shown in Fig. 5a and b, demonstrating the ability of the multi-architecture to yield high local-field confinement. All simulated electric and magnetic field intensity maps were normalized with respect to the field amplitudes obtained from a reference air–substrate interface under identical excitation conditions. This normalization allows direct comparison across the different photonic–plasmonic architectures studied and provides a consistent quantitative framework for assessing the relative field enhancement and emission behavior. The cryosoret nano-assemblies render circulating electric flux that in turn produce magnetic flux thereby utilizing the full potential of light energy, which is further augmented with the TM and TE resonances of the PC substrate. Moreover, the simulations of a radiating dipole at such interfaces show an increase in the far-field radiation (Fig. 5c and d). We note all simulations presented in this work were performed for a specific dipole orientation (*x*-, *y*-, *z*-oriented) and planar configuration of the photonic–plasmonic stack. Dipole orientation averaging was not considered, and hence the reported fluorescence enhancement values may vary under random dipole distributions that more closely represent experimental conditions. Similarly, the possible influence of planar anisotropy arising from the two-dimensional grating structure and layered architecture (PC–AuNP–hBN–Au cryosorets) was not explicitly analyzed. These aspects such as the effect of dipole orientation averaging and azimuthal anisotropy, are expected to provide further mechanistic insights into the emission behavior and will be systematically investigated in the future.

**Fig. 5:**
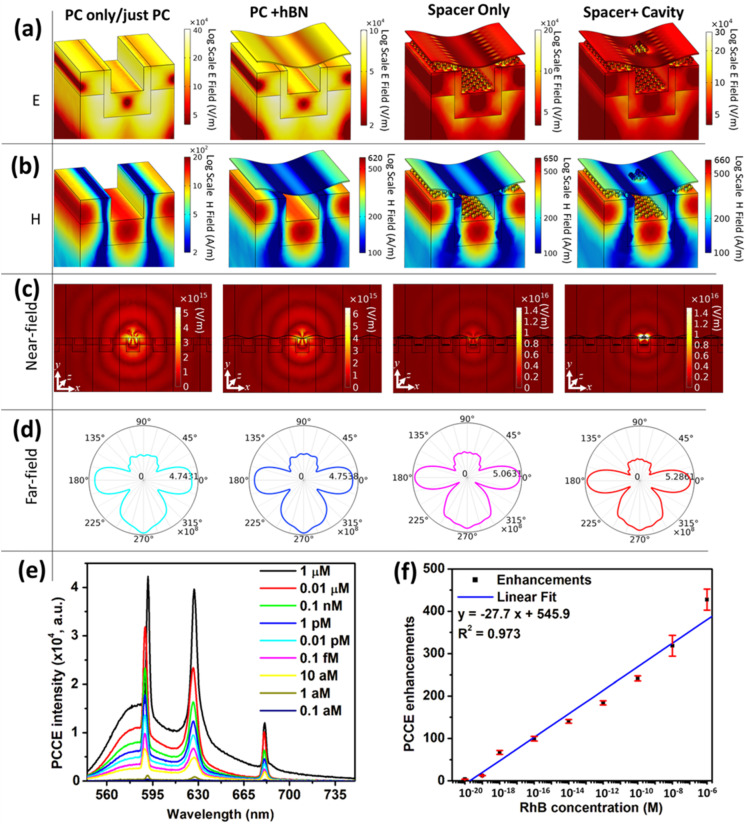
Simulations showing electric and magnetic hotspots and experimental sensing. COMSOL Multiphysics simulations showing the calculated (a) electric field distribution and (b) magnetic field distribution of different interfaces, namely PC, PC + hBN, PC + AuNP + hBN, PC + AuNP + hBN + AuCS for TM polarization (TE polarization is presented in Fig. S7). The simulation parameters are presented in the SI. The size, shape and geometry of the nanostructures are taken from AFM and SEM measurements. Here, all the electric and magnetic field intensities shown are not normalized. They can be normalized to the incident field intensity values: *E*_0_ = 2.7 × 10^4^ V m^−1^ and *H*_0_ = 73 A m^−1^, respectively. (c) Calculated near-field distributions and (d) far-field radiation pattern of radiating dipole (*x*-oriented) when interfaced with different configurations. (e) PCCE intensity and (f) PCCE enhancements obtained by using different concentrations of the radiating dipoles.

The hybrid spacer + cavity nanointerface that yielded the highest fluorescence enhancements were utilized for the detection of RhB fluorescent reporter. The performance of the sensor platform was evaluated by linearly decreasing the concentration of the emitter from 1 µM to 0.01 aM and incorporating it onto the PCEF platform. The corresponding spectral intensity plots for different concentrations are shown in Fig. 5e. The emission enhancement (plotted in Fig. 5f) was calculated using eqn (1), defined as the ratio of the fluorescence intensity obtained on the photonic crystal (PC) substrate to that measured on the glass substrate at the emission maximum wavelength for the given sample. This approach is consistent with methodologies adopted in previous studies.^21,61,69,75,76^ Comparison of the hybrid photonic-plasmonic systems, fluorescence enhancement, analyte detected, and limit of detection with the literature are summarized in Table S1 (SI). As shown in Fig. 5e, we observe that the fluorescence spectral intensity decreased with the decrease in the concentration of the emitter where the PC coupled emission becomes negligible at concentrations less than 1 aM. The emission enhancements are plotted in Fig. 5f showing a long linear range (10 orders of magnitude) from 1 µM to 1 aM (LOD). This high sensor performance is attributed to the prevention of the direct detrimental quenching pathways rendered by the hBN spacer nanolayer yet being able to preserve the near-field optical coupling, in addition to the EM field amplification fostered by the cryosorets driven cavity.

Our approach realizes the widely investigated concept of metal–dielectric–metal (MDM) interfaces in the field of plasmonics, where strong coupling boosts fluorescence emission through the Purcell effect.^77^ To the best of our knowledge, this is the first successful demonstration of integrating such a hybrid PC + AuNP + hBN + fluorophore + AuCS architecture with an ultrathin hBN spacer to achieve optimized fluorescence enhancement with a different number of NPs per assembly of AuCS. The synergistic effects observed in our architecture result from the simultaneous utilization of high-Q PC modes, strong near-field plasmonic enhancements from AuNPs, HRI dielectric hBN spacer, and engineered plasmonic cavities formed by the nanoassemblies.

## Author contributions

S. Bhattacharya and S. Bhaskar contributed equally to this work. S. Bhattacharya: conceptualization, data curation, formal analysis, investigation, methodology, software; validation, visualization, writing – original draft, writing – review & editing; S. Bhaskar: conceptualization, data curation, formal analysis, funding acquisition, investigation, methodology, software; validation, visualization, writing – original draft, writing – review & editing; W. Liu: conceptualization, data curation, formal analysis, investigation, software; validation, visualization, writing – original draft; J. Tibbs: data curation, formal analysis, methodology, software, visualization; V. Pachchigar: data curation, formal analysis, methodology, software, visualization; R. M. Sankaran: conceptualization, funding acquisition, project administration, resources, supervision, validation, visualization, writing – review & editing; B. T. Cunningham: conceptualization, funding acquisition, project administration, resources, supervision, validation, visualization, writing – review & editing.

## Conflicts of interest

There are no conflicts to declare.

## Supplementary Material

NR-017-D5NR02950C-s001

## Data Availability

The data supporting this article have been included as part of the supplementary information (SI). Supplementary information is available. See DOI: https://doi.org/10.1039/d5nr02950c.

## References

[cit1] Inan H., Poyraz M., Inci F., Lifson M. A., Baday M., Cunningham B. T., Demirci U. (2017). Chem. Soc. Rev..

[cit2] Li N., Canady T. D., Huang Q., Wang X., Fried G. A., Cunningham B. T. (2021). Nat. Commun..

[cit3] Shafiee H., Lidstone E. A., Jahangir M., Inci F., Hanhauser E., Henrich T. J., Kuritzkes D. R., Cunningham B. T., Demirci U. (2014). Sci. Rep..

[cit4] Borghei Y.-S., Hosseinkhani S., Ganjali M. R. (2022). J. Adv. Res..

[cit5] Wang M., Zhou F., Lu X., McClung A., Davanco M., Aksyuk V. A., Srinivasan K. (2022). Phys. Rev. Lett..

[cit6] Bhaskar S., Srinivasan V., Ramamurthy S. S. (2023). ACS Appl. Nano Mater..

[cit7] Zengin G., Wersäll M., Nilsson S., Antosiewicz T. J., Käll M., Shegai T. (2015). Phys. Rev. Lett..

[cit8] Yang J., Zhang H., Wang T., De Leon I., Zaccaria R. P., Qian H., Chen H., Wang G. (2022). Phys. Rev. Appl..

[cit9] Al-Rashid A., John S. (2015). Phys. Rev. Appl..

[cit10] Rai B., Malmberg R., Srinivasan V., Ganesh K. M., Kambhampati N. S. V., Andar A., Rao G., Sanjeevi C. B., Venkatesan K., Ramamurthy S. S. (2021). ACS Sens..

[cit11] Bhaskar S., Das P., Moronshing M., Rai A., Subramaniam C., Bhaktha S. B. N., Ramamurthy S. S. (2021). Nanophotonics.

[cit12] Cheerala V. S. K., Ganesh K. M., Bhaskar S., Ramamurthy S. S., Neelakantan S. C. (2023). Langmuir.

[cit13] Faggiani R., Yang J., Lalanne P. (2015). ACS Photonics.

[cit14] Kongsuwan N., Demetriadou A., Chikkaraddy R., Benz F., Turek V. A., Keyser U. F., Baumberg J. J., Hess O. (2018). ACS Photonics.

[cit15] Dulkeith E., Morteani A. C., Niedereichholz T., Klar T. A., Feldmann J., Levi S. A., van Veggel F. C. J. M., Reinhoudt D. N., Möller M., Gittins D. I. (2002). Phys. Rev. Lett..

[cit16] Lakowicz J. R. (2005). Anal. Biochem..

[cit17] Anger P., Bharadwaj P., Novotny L. (2006). Phys. Rev. Lett..

[cit18] Yokota H., Saito K., Yanagida T. (1998). Phys. Rev. Lett..

[cit19] Bhaskar S., Kowshik N. C. S. S., Chandran S. P., Ramamurthy S. S. (2020). Langmuir.

[cit20] Bhaskar S., Liu L., Liu W., Tibbs J., Akin L. D., Bacon A., Cunningham B. T. (2025). APL Mater..

[cit21] Bhaskar S., Liu L., Liu W., Tibbs J., Cunningham B. T. (2025). MRS Bull..

[cit22] Rai A., Bhaskar S., Ganesh K. M., Ramamurthy S. S. (2022). Mater. Chem. Phys..

[cit23] de Leon N. P., Shields B. J., Yu C. L., Englund D. E., Akimov A. V., Lukin M. D., Park H. (2012). Phys. Rev. Lett..

[cit24] Sauvan C., Hugonin J. P., Maksymov I. S., Lalanne P. (2013). Phys. Rev. Lett..

[cit25] Barya P., Xiong Y., Shepherd S., Gupta R., Akin L. D., Tibbs J., Lee H., Singamaneni S., Cunningham B. T. (2023). Small.

[cit26] Chaudhery V., George S., Lu M., Pokhriyal A., Cunningham B. T. (2013). Sensors.

[cit27] Chen W., Long K. D., Yu H., Tan Y., Sun Choi J., Harley B. A., Cunningham B. T. (2014). Analyst.

[cit28] Cunningham B. T. , Frontiers in Pathogen Detection: From Nanosensors to Systems, 2010

[cit29] Huang C.-S., George S., Lu M., Chaudhery V., Tan R., Zangar R. C., Cunningham B. T. (2011). Anal. Chem..

[cit30] Kinkhabwala A., Yu Z., Fan S., Avlasevich Y., Müllen K., Moerner W. E. (2009). Nat. Photonics.

[cit31] Novotny L., van Hulst N. (2011). Nat. Photonics.

[cit32] Ringler M., Schwemer A., Wunderlich M., Nichtl A., Kürzinger K., Klar T. A., Feldmann J. (2008). Phys. Rev. Lett..

[cit33] Li J.-F., Li C.-Y., Aroca R. F. (2017). Chem. Soc. Rev..

[cit34] Yao J., Yang M., Duan Y. (2014). Chem. Rev..

[cit35] Gan W., Tserkezis C., Cai Q., Falin A., Mateti S., Nguyen M., Aharonovich I., Watanabe K., Taniguchi T., Huang F., Song L., Kong L., Chen Y., Li L. H. (2019). ACS Nano.

[cit36] Yi M., Zhang D., Wen X., Fu Q., Wang P., Lu Y., Ming H. (2011). Plasmonics.

[cit37] Saboktakin M., Ye X., Oh S. J., Hong S.-H., Fafarman A. T., Chettiar U. K., Engheta N., Murray C. B., Kagan C. R. (2012). ACS Nano.

[cit38] Mertens J., Eiden A. L., Sigle D. O., Huang F., Lombardo A., Sun Z., Sundaram R. S., Colli A., Tserkezis C., Aizpurua J., Milana S., Ferrari A. C., Baumberg J. J. (2013). Nano Lett..

[cit39] Hwang S. W., Shin D. H., Kim C. O., Hong S. H., Kim M. C., Kim J., Lim K. Y., Kim S., Choi S.-H., Ahn K. J., Kim G., Sim S. H., Hong B. H. (2010). Phys. Rev. Lett..

[cit40] Kim G., Kim M., Hyun C., Hong S., Ma K. Y., Shin H. S., Lim H. (2016). ACS Nano.

[cit41] Tran T. T., Bray K., Ford M. J., Toth M., Aharonovich I. (2016). Nat. Nanotechnol..

[cit42] Dowran M., Kilic U., Lamichhane S., Erickson A., Barker J., Schubert M., Liou S.-H., Argyropoulos C., Laraoui A. (2025). Laser Photonics Rev..

[cit43] Jang J., Jeong M., Lee J., Kim S., Yun H., Rho J. (2023). Adv. Mater..

[cit44] Genc S. , Yucel O., Aglarci F., Rodriguez-Fernandez C., Yilmaz A., Caglayan H., Ates S. and Bek A., arXiv, 2025, preprint, arXiv:2506.14517, 10.48550/arXiv.2506.14517

[cit45] Kim S., Fröch J. E., Gardner A., Li C., Aharonovich I., Solntsev A. S. (2019). Opt. Lett..

[cit46] Li P., Dolado I., Alfaro-Mozaz F. J., Casanova F., Hueso L. E., Liu S., Edgar J. H., Nikitin A. Y., Vélez S., Hillenbrand R. (2018). Science.

[cit47] Dowran M., Butler A., Lamichhane S., Erickson A., Kilic U., Liou S. H., Argyropoulos C., Laraoui A. (2023). Adv. Opt. Mater..

[cit48] Titov M., Katsnelson M. I. (2014). Phys. Rev. Lett..

[cit49] Grudinin D. V., Ermolaev G. A., Baranov D. G., Toksumakov A. N., Voronin K. V., Slavich A. S., Vyshnevyy A. A., Mazitov A. B., Kruglov I. A., Ghazaryan D. A., Arsenin A. V., Novoselov K. S., Volkov V. S. (2023). Mater. Horiz..

[cit50] Liu L., Bhaskar S., Cunningham B. T. (2024). Appl. Phys. Lett..

[cit51] Rai A., Bhaskar S., Ganesh K. M., Ramamurthy S. S. (2022). ACS Appl. Nano Mater..

[cit52] Flauraud V., Regmi R., Winkler P. M., Alexander D. T. L., Rigneault H., van Hulst N. F., García-Parajo M. F., Wenger J., Brugger J. (2017). Nano Lett..

[cit53] Alù A., Engheta N. (2009). Phys. Rev. Lett..

[cit54] Bouchet D., Cao D., Carminati R., De Wilde Y., Krachmalnicoff V. (2016). Phys. Rev. Lett..

[cit55] Moronshing M., Subramaniam C. (2018). ACS Sustainable Chem. Eng..

[cit56] Mondal S., Subramaniam C. (2020). ACS Sustainable Chem. Eng..

[cit57] Moronshing M., Bhaskar S., Mondal S., Ramamurthy S. S., Subramaniam C. (2019). J. Raman Spectrosc..

[cit58] Yuan Y., Weber J., Li J., Tian B., Ma Y., Zhang X., Taniguchi T., Watanabe K., Lanza M. (2024). Nat. Commun..

[cit59] Ma K. Y., Zhang L., Jin S., Wang Y., Yoon S. I., Hwang H., Oh J., Jeong D. S., Wang M., Chatterjee S., Kim G., Jang A. R., Yang J., Ryu S., Jeong H. Y., Ruoff R. S., Chhowalla M., Ding F., Shin H. S. (2022). Nature.

[cit60] Kirchhoff A., Deilmann T., Krüger P., Rohlfing M. (2022). Phys. Rev. B.

[cit61] Bhaskar S., Liu W., Tibbs J., Cunningham B. T. (2024). Appl. Phys. Lett..

[cit62] Bhaskar S., Das P., Srinivasan V., Bhaktha S. B. N., Ramamurthy S. S. (2022). Mater. Res. Bull..

[cit63] Bhaskar S., Singh A. K., Das P., Jana P., Kanvah S., Bhaktha B. N. S., Ramamurthy S. S. (2020). ACS Appl. Mater. Interfaces.

[cit64] Bhaskar S., Lis S. M., Kanvah S., Bhaktha B. N. S., Ramamurthy S. S. (2023). ACS Appl. Opt. Mater..

[cit65] Lis S. M. S, Bhaskar S., Dahiwadkar R., Kanvah S., Ramamurthy S. S., Bhaktha B. N. S. (2023). ACS Appl. Nano Mater..

[cit66] Anker J. N., Hall W. P., Lyandres O., Shah N. C., Zhao J., Van Duyne R. P. (2008). Nat. Mater..

[cit67] Mejía-Salazar J. R., Oliveira Jr O. N. (2018). Chem. Rev..

[cit68] Bhaskar S. (2023). Micromachines.

[cit69] Bhaskar S., Liu L., Liu W., Tibbs J., Akin L. D., Bacon A., Cunningham B. T. (2025). APL Mater..

[cit70] Cheerala V. S. K., Ganesh K. M., Bhaskar S., Ramamurthy S. S., Neelakantan S. C. (2023). Langmuir.

[cit71] Kelf T. A., Sugawara Y., Cole R. M., Baumberg J. J., Abdelsalam M. E., Cintra S., Mahajan S., Russell A. E., Bartlett P. N. (2006). Phys. Rev. B:Condens. Matter Mater. Phys..

[cit72] Baumberg J. J., Aizpurua J., Mikkelsen M. H., Smith D. R. (2019). Nat. Mater..

[cit73] Pavaskar P., Theiss J., Cronin S. B. (2012). Opt. Express.

[cit74] Yang J., Faggiani R., Lalanne P. (2016). Nanoscale Horiz..

[cit75] Xiong Y., Shepherd S., Tibbs J., Bacon A., Liu W., Akin L. D., Ayupova T., Bhaskar S., Cunningham B. T. (2023). Micromachines.

[cit76] Bhaskar S., Liu W., Shepherd S., Wijewardena D., Tibbs J., Cunningham B. T. (2025). Adv. Funct. Mater..

[cit77] Chikkaraddy R., de Nijs B., Benz F., Barrow S. J., Scherman O. A., Rosta E., Demetriadou A., Fox P., Hess O., Baumberg J. J. (2016). Nature.

