# Supplementary material for: Photonic crystal enhanced fluorescence using a hybrid hexagonal boron nitride spacer and plasmonic gold cryosoret cavity
Source: Nanoscale. 2025 Nov 5;17(46):26678–88. doi: 10.1039/d5nr02950c (PMC12606722; doi:10.1039/d5nr02950c)
Supplement: NR-017-D5NR02950C-s001 [file NR-017-D5NR02950C-s001.pdf]

Supplementary Information (SI) for Nanoscale.
This journal is © The Royal Society of Chemistry 2025

Supplementary information

Photonic Crystal Enhanced Fluorescence using a Hybrid Hexagonal Boron Nitride Spacer and Plasmonic Gold Cryosoret Cavity

Souvik Bhattacharya,[1,2#] Seemesh Bhaskar,[3,4,5#] Weinan Liu,[3,4] Joseph Tibbs,[4,5] Vivek Pachchigar,[1,2] R. Mohan Sankaran,[1,2*] and Brian T. Cunningham[3,4,5,6,7,8*]

[1]Department of Nuclear, Plasma, and Radiological Engineering, The Grainger College of Engineering, University of Illinois Urbana-Champaign, Champaign, IL, USA
[2]Materials Research Laboratory, University of Illinois Urbana-Champaign, Urbana, IL, USA
[3]Department of Electrical and Computer Engineering, University of Illinois Urbana-Champaign, Urbana, IL 61801, USA
[4]Nick Holonyak Jr. Micro and Nanotechnology Laboratory, University of Illinois Urbana-Champaign, Urbana, IL 61801, USA
[5]Carl R. Woese Institute for Genomic Biology, University of Illinois Urbana-Champaign, Urbana, IL 61801, USA
[6]Department of Bioengineering, University of Illinois at Urbana-Champaign, Urbana, IL 61801, USA
[7]Department of Chemistry, University of Illinois Urbana-Champaign, Urbana, IL 61801, USA
[8]Cancer Center at Illinois, Urbana, IL 61801, USA

Number of Figures: 8 (S1 to S8)

Number of Tables: 1

Number of pages: 18

Table of contents:

**Sl. No.**	**Table of content**	**Page number**
1	Growth of large-area, continuous hBN films	S2
2	Film transfer methodology	S2
3	Characterization of hBN films	S2
4	A brief overview of the radiating plasmon model	S3
5	Radiating Guided Mode Resonance (GMR) Model: Polarization and Fluorescence Insights (Figure S1)	S3
6	Polarization property of the PC coupled emission (Figure S2)	S4
7	Scanning electron microscopy at different resolutions (Figures S3, S4, S5)	S7-S9
8	Lifetime measurements of different samples (Figure S6)	S10
9	COMSOL Multiphysics simulations (Figure S7)	S11
10	Future scope and perspectives (Figure S8)	S11-S14
11	Comparative analysis of the hybrid photonic-plasmonic systems, fluorescence enhancement, analyte detected, and limit of detection from different works with our current research work	S16-S17
12	References	S18-S22

Growth of large-area, continuous hBN films

hBN films were grown by low-pressure, hot-wall chemical vapor deposition using a Lindberg Blue M furnace. Prior to growth, the system was pumped down to achieve a base pressure of ~8 mTorr using a dry scroll pump. Substrates for growth were 25 µm thick Cu foil (ESPI Metals). Before growth, Cu substrates 1.5 cm x 1.5 cm in size were pre-cleaned by sonicating in acetone and IPA, followed by a dip in 0.5 M HCl to strip the oxide layer. The substrates were then placed on a ceramic holder in the center of a high purity 2 in. fused quartz tube (Technical Glass Products Inc.). Roughly 30 mg of ammonia borane (Boron Specialties, 99%) was placed in a sealed stainless-steel ampule upstream of the substrate. The system was then pumped down to base pressure and purged with a flow of 500 sccm Ar. The gas flows were then switched to 300 sccm Ar and 100 sccm H_2, and the furnace was ramped to 1030 ^oC and held for 2 h to anneal the Cu substrate and facilitate grain growth. The ammonia borane ampule was heated to ~85 ^oC for 30 min. Following the annealing step, the valve isolating the precursor was opened for 20 min to initiate hBN growth and then closed to stop further unwanted deposition. The sample was then cooled at 5 ^oC/min to 650 ^oC after which it was rapidly brought down to room temperature.

Film transfer methodology

hBN films were transferred from Cu substrates using a PMMA-assisted wet transfer method. Briefly, a PMMA solution (A8, Micro Chem) was deposited on hBN/Cu by spin coating at 4000 RPM for 30 s, followed by baking at 90 ^oC for 10 min to eliminate any residual solvent. The sample was then placed in a solution of 0.2 M ammonium persulfate (APS, Sigma) to completely dissolve the Cu which typically took overnight. The free floating PMMA/hBN film was then scooped onto a SiO_2/Si wafer, PC, or Au/PC and heated at 110 ^oC for 30 min to promote adhesion of the hBN. Finally, PMMA was dissolved by immersing the sample in acetone overnight.

Characterization of hBN films

hBN films were characterized after transferring the film from the Cu substrate to an appropriate substrate (SiO_2, fused silica, or TEM grids) compatible with the type of technique used. Raman spectroscopy and mapping were performed using a Nanophoton Raman 11 instrument with a 532 nm green laser focused through a 50x objective lens (0.8 NA). Spectra were acquired with a 1200 gr/mm grating leading to 1 cm^{-1} spectral resolution or better. AFM measurements were performed

in tapping mode using an Asylum Cypher scanning probe microscope and Al-coated Si cantilevers (NanoWorld, ARROW-NCR, 160 um) exhibiting a resonance frequency of 285 kHz. XPS spectra were acquired using the Ion Gas Neutral Interaction with Surfaces (IGNIS) facility. An Mg K-alpha source (Specs Surface Nano Analysis GmbH) emitting 1253.7 eV X-rays was used for all measurements. The pass energy was set to 60 eV for survey spectra and 40 eV for high-resolution scanning. UV-Vis absorption spectra were recorded using a Varian Cary 5G spectrophotometer in the range of 190 to 1000 nm by transferring the hBN onto UV-transparent fused silica. A clean, bare piece of fused silica was used as the reference for all absorption measurements. SEM images were acquired using a Hitachi S-4800 high resolution SEM following sputtering using a Emscope SC 500. HRTEM images were obtained using a JEOL 2100 TEM on hBN films transferred onto quantifoil TEM grids.

Radiating plasmon model

The radiating plasmon model (RPM) was first established by Lakowicz et al. in the early 2000s [1-4], marking the seminal advancement of the broad field of plasmon-enhanced fluorescence (PEF) [5-9] which is also known as metal-enhanced fluorescence (MEF) as well as surface plasmon-coupled emission (SPCE) [10-14]. The concepts of RPM explained the far-field and near-field radiation characteristics of an emitter (fluorescent molecules, quantum dots and nanodiamonds) that is in close proximity to a metallic species (metallic nanoparticles, island films or continuous films) [10, 15, 16]. In contrast to the traditional fluorescence based understanding the RPM reshaped the way researchers understood the emission attributes where polarization, quantum yield, lifetime and collection efficiency were considered as key parameters that determines the global fluorescence output. Fundamentally, as per the RPM the surface plasmon polaritons (SPPs) on metallic thin films (as well as localized surface plasmon resonances on metallic nanomaterials) can be excited by the emission itself (and not the laser / excitation source) and these excited plasmons can radiate to the far field with specific polarization and directional attributes (when the phase matching and boundary conditions are satisfied) [1, 3]. This is accompanied with reduction in fluorescence lifetime (and increase photostability and quantum yield) due to additional pathways of radiative decay rendered by plasmonic NPs. Moreover, the far field spectral signal resembles that of the fluorophore with the polarization attributes being that of the plasmonic systems. Consequently, the collection efficiency and the fluorescence signal intensity is dramatically

enhanced making it a potential platform for biosensing applications [13, 15, 17-22]. However, the major drawback of these systems includes the dependence on prism-based Kretschmann configurations to achieve phase-matching conditions as well as the detrimental losses observed in the plasmonic systems which inevitably generate non-radiative decay pathways (resulting in surface induced quenching effects). In order to overcome these drawbacks researchers explored alternative and complementary models that retains the best outcomes of the plasmonic systems while at the same time mitigating losses and enabling prism-free architectures [15, 23-25].

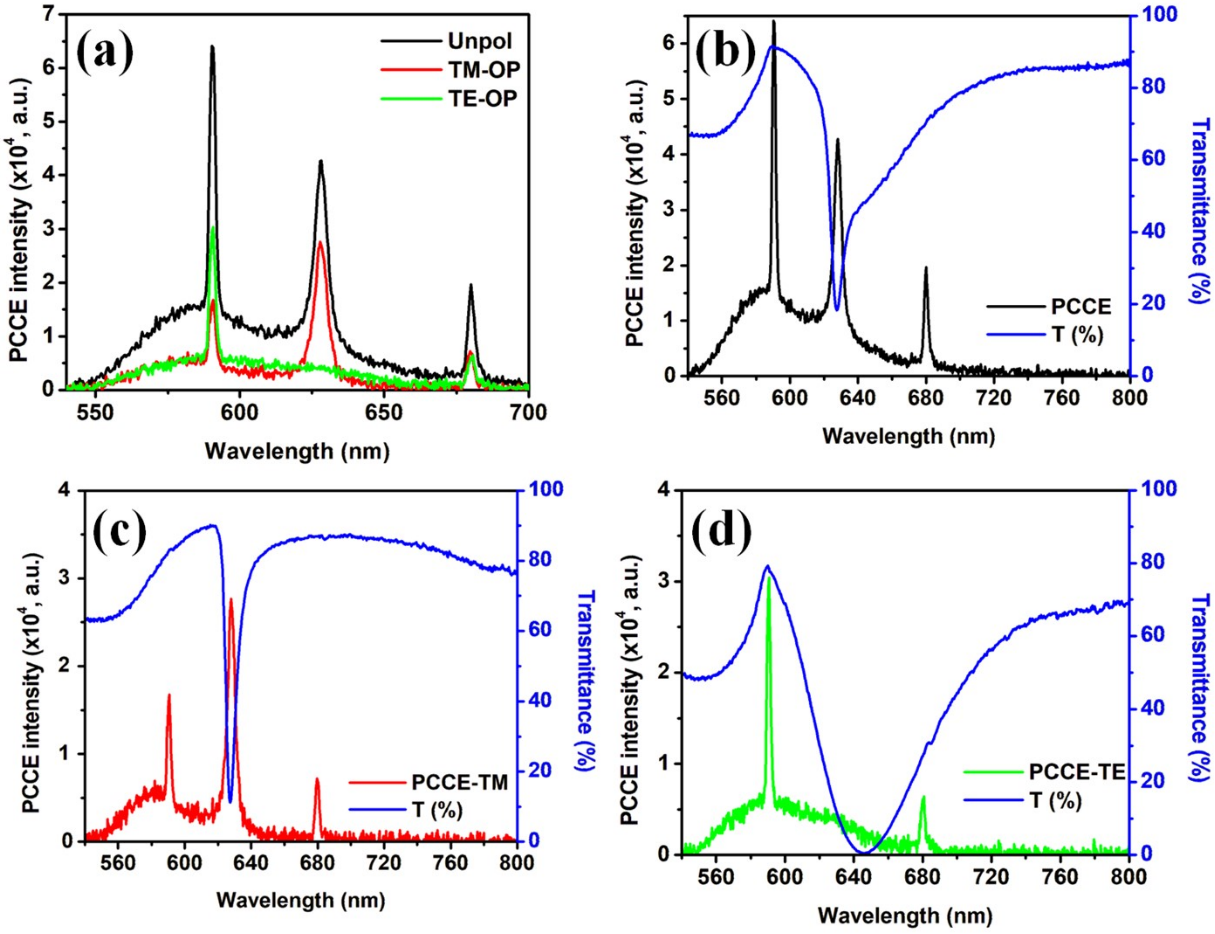

Figure S1. Photonic crystal-coupled emission (PCCE) and transmittance measurements. (a) PCCE spectra for unpolarized (unpol), TM-polarized and TE-polarized. OP means output, where the polarizer is placed after the emission is coupled to the PC substrate. That is, the polarizer is in the output region in between the PC substrate and the detector. Overlap of the experimental Transmittance (T) spectra and fluorescence data obtained for (b) unpolarized, (c) TM polarized and (d) TE polarized measurements.

Radiating guided mode resonance (GMR) model

In the year 2024, our group demonstrated the emergence of steering emission in a prism-free, metal-free and objective-free platform by careful engineering of the photonic crystal - radiating dipole interface [25-27]. We proposed the radiating guided mode resonance (GMR) model to explain the light-coupling phenomena that was observed experimentally and theoretically. It is worth noting that our work is inspired by the pioneering research by Lakowicz and co-workers in the broad domain of plasmon-controlled fluorescence as well as models like RPM (explained in previous section of this document). Fundamentally, the photonic crystals (PCs) can be strategically designed to support GMRs which are optical modes that can selectively couple to the incident laser light or emitted light from the adjacent radiating dipole based on factors such as wavelength of resonance, polarization, and angular orientation of the excitation (source) and emission (detector) [26, 28-33]. Similar to the RPM, when a radiating dipole is placed over a PC substrate and excited using a laser source, the emitted photons (of the fluorophores) can excite the GMR which can then re-radiate to the far field carrying the properties of the fluorescence spectral attributes and polarization selectivity of the PC substrate (analogues to how plasmons radiate in RPM) in a lossless substrate [25-27]. While the Photonic crystal enhanced fluorescence (PCEF) is a very broad classification, photonic crystal-coupled emission (PCCE) can be understood as a sub-classification of PCEF [34]. In our earlier works we demonstrated that the PCCE can be enhanced using transverse electric (TE) as well as transverse magnetic (TM) polarized emission collection, while such collection of both polarizations is not possible in purely plasmonic systems (as they couple to TM modes only) [3, 35-37]. As shown in the simulated and experimental dispersion diagrams (Figure 3 of the manuscript) the resonance of the PC is tuned to match the emission maximum of the fluorophore (rhodamine B) under consideration. Consequently, the emission is coupled to the underlying modes sustained by the PC substrates and gets substantially enhanced while reaching the detector in the far-field. In our experiments we observed that the maximum fluorescence enhancement was observed when the GMR of the PC matched the emission peak of the fluorophores (and not necessarily the excitation wavelength). Such a system presents more flexibility and optimized design as the PCEF measurements now does not depend of careful excitation of the GMR in the first place [26, 28, 32-34]. Moreover, this method also presents a departure from conventional SPCE and coupled emission in the case of Bragg mirrors (sustaining Bloch surface waves and internal optical modes) that depend on the excitation optics relying on bulky and prism dependent systems [10, 15, 24, 38, 39].

Figure S1 presents the unpolarized, TM-polarized and TE-polarized data for the sample configuration that yielded the highest fluorescence enhancements. We note that there is an excellent overlap between the modes sustained by the PC and the emission spectral profile that is allowed by the PC (or in other words, the PCCE). The enhanced emission observed for the hybrid system of PC+AuNP+hBN+AuCS is attributed to the increased local density of optical states (LDOS) at the emission window which results in lesser lifetime and enhanced photostability (as the emitter spends less time in the excited state) [1, 3, 40, 41]. Our experimental fluorescence and transmittance measurements are cross-validated using rigorous coupled-wave analysis (RCWA) and COMSOL simulations (detailed in the manuscript). For all our simulations, we used the built-in COMSOL boron nitride material[42]. Additionally, our experimental and simulation results present increase in the coupling efficiency with the incorporation of plasmonic nano-assemblies (cryosorets) [10, 25, 26, 43-45]. This is attributed to the 3D-distributed nanogaps, creating ultra-intense "hotspots" that magnify both excitation and emission processes. Additionally, the anisotropic arrangement of nanoparticles leads to a multiplicative "cascading" of local fields, increasing both near-field intensity and far-field emission functionality in the presence of a radiating dipole [46-50]. When combined with the PCCE systems, these structures dequench the previously quenched dipoles and simultaneously steer the emission through GMR modes hence presenting a powerful synergy of plasmonic (RGM) and photonic effects (radiating GMR).

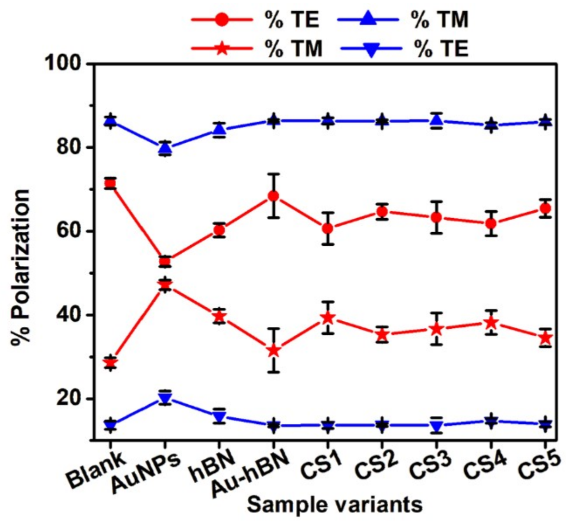

Figure S2. Percentage polarization observed for photonic crystal-coupled emission (PCCE) obtained experimentally for different sample variants. The blue lines correspond to the GMR coupled emission and the red colored data correspond to the Band edge coupled emission.

Further, the percentage polarization calculated as %p = 100* p/(p+s) for the fluorescence spectral intensities obtained for different samples are shown in Figure S2. We note that the PCCE is highly polarized hence indicating the experimental data being in accordance with the radiating GMR model [25-27, 51]. We note that the AuNPs that resulted in substantial quenching showed very less polarization selectivity because of the surface-induced quenching effects dominating the PC coupled emission property [15, 17, 25, 52, 53].

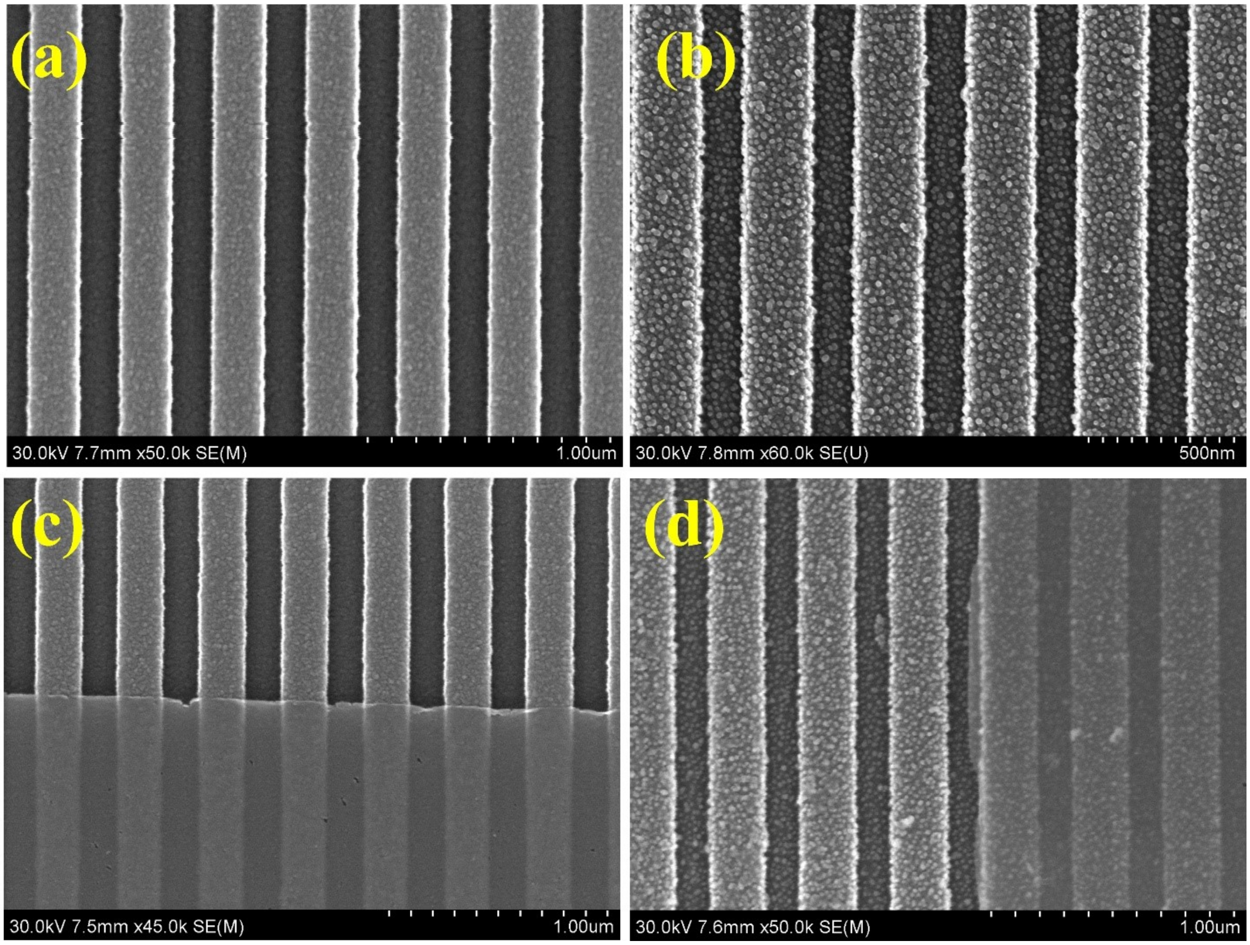

Figure S3. SEM images of (a) PC, (b) PC+AuNPs, (c) PC+hBN and (d) PC+AuNP+hBN at low resolution (magnification ~50k).

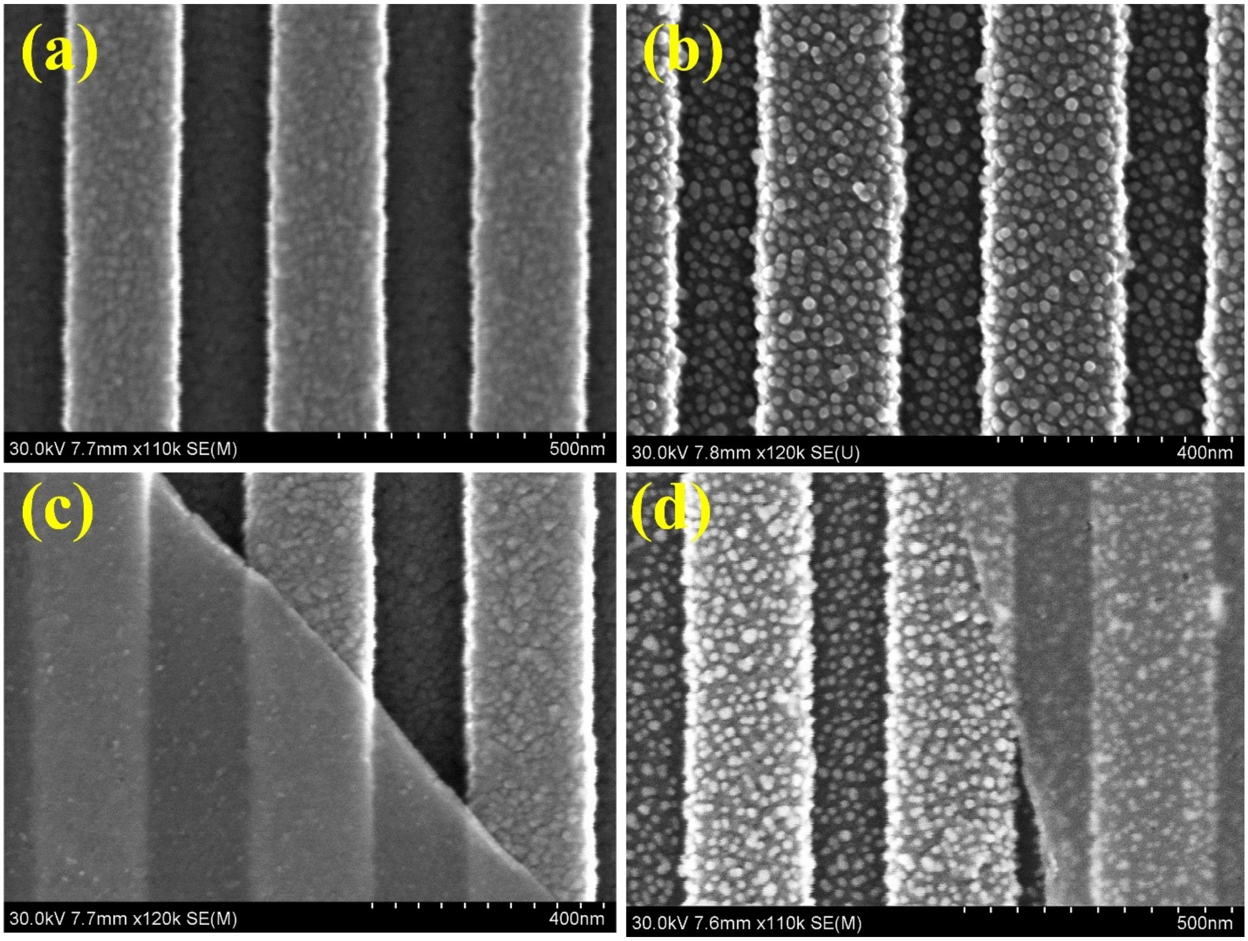

Figure S4. SEM images of (a) PC, (b) PC+AuNPs, (c) PC+hBN and (d) PC+AuNP+hBN at moderate resolution (magnification ~120k).

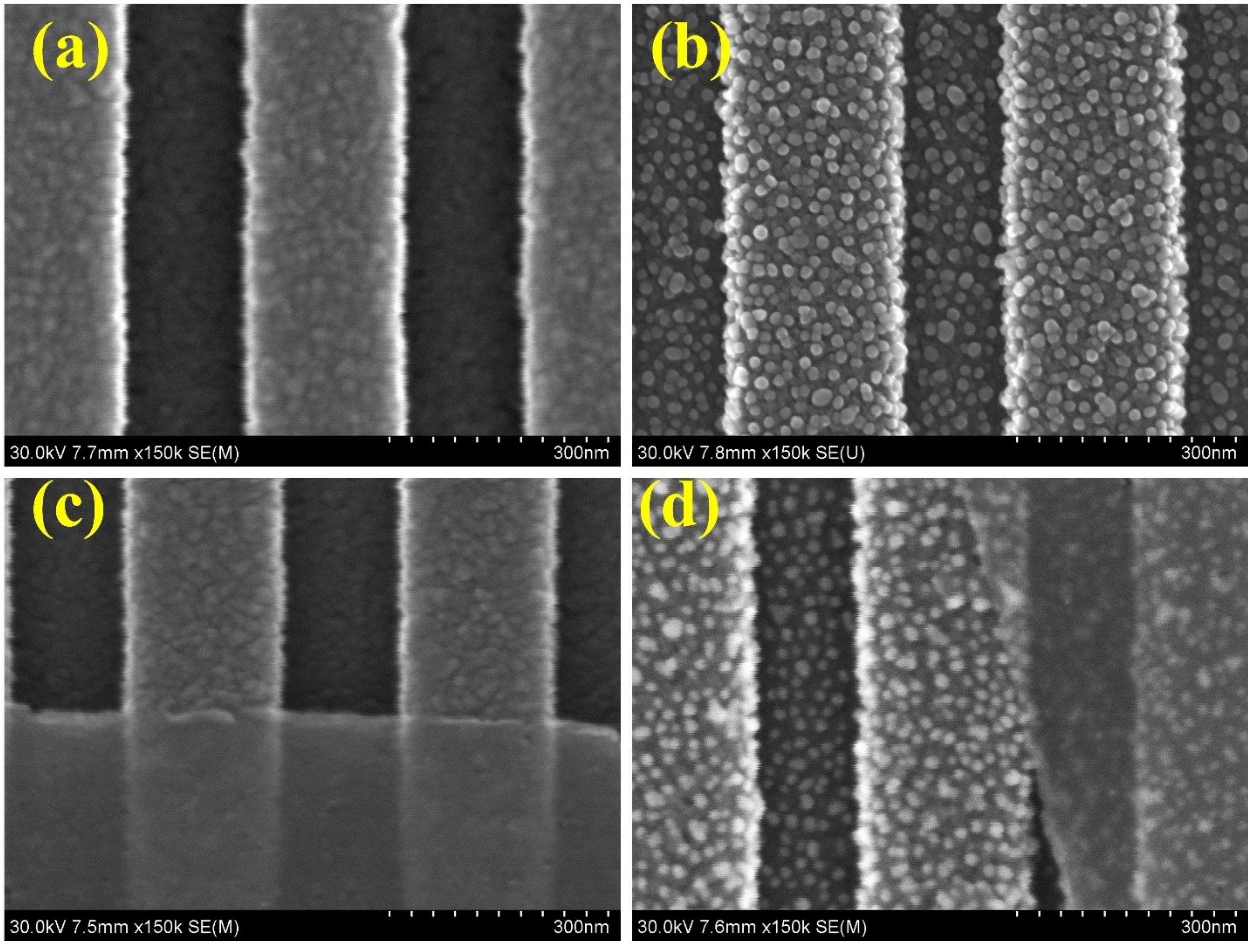

Figure S5. SEM images of (a) PC, (b) PC+AuNPs, (c) PC+hBN and (d) PC+AuNP+hBN at high resolution (magnification ~150k).

3. Hybrid interface with hBN spacer and cryosoret cavity engineering

Our work further elaborates on the utility of hybrid platform by incorporating hBN as a 2D nanospacer between the gold monolayer (on PC) and dye molecules (rhodamine B). On account of its high chemical stability, large bandgap, and low-loss optical attributes, they serve as ideal interlayer spacer for controlling the radiative and non-radiative energy transfer processes [54, 55]. While the spacer layer assists in avoiding the quenching effects, on account of its very thin structural property it also yields room for sufficient near-field overlap for field enhancement. While the pristine PC showed 2.11 ns lifetime for the radiating dipoles, the addition of hBN resulted in reduction of the lifetime to 1.05 ns, 64.1% and 2.72 ns, 35.9%. This biexponential nature can be attributed to the two different types of near-field effects experienced by the radiating dipoles (GMR of PC and HRI hBN) [56-58]. Similarly, a biexponential decay presenting 1.68 ns,

65.9% and 2.77 ns, 34.1% was observed for PC+AuNPs system. Also, adding the hBN coating on top of the PC+AuNP hybrid resulted in triexponential decay (1.00 ns, 59.2%; 2.32 ns, 11.9% and 2.90 ns, 28.9%), once again due to the influence of three components in determining the local field intensity [57, 58].

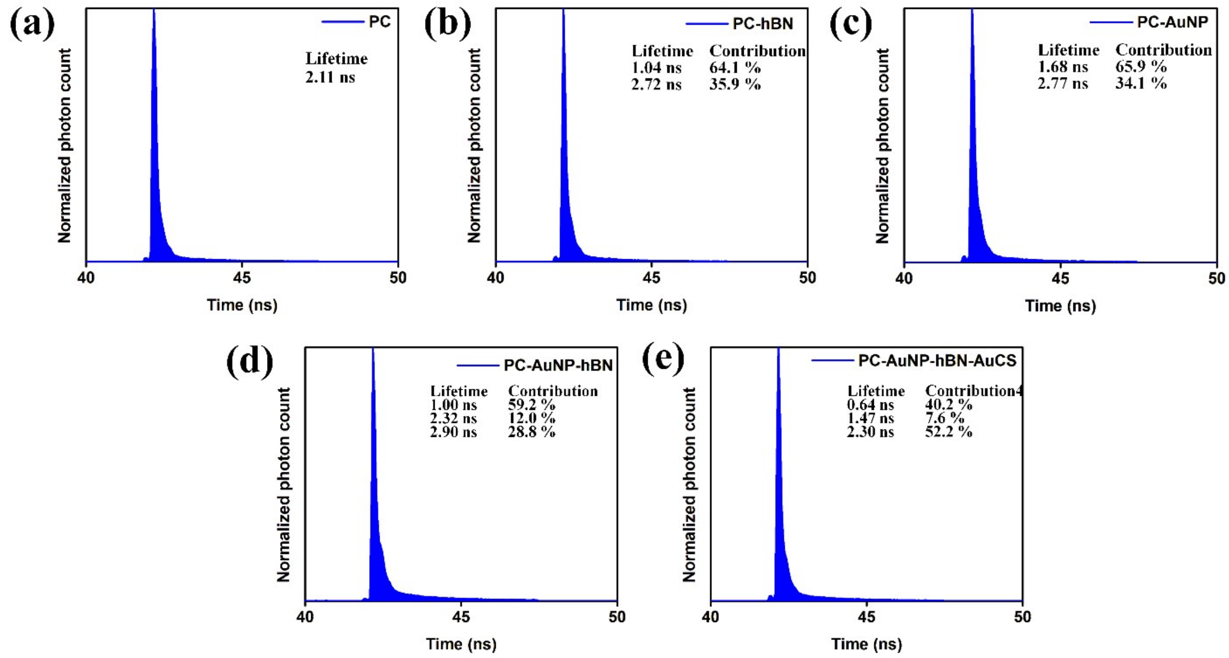

Figure S6. Experimental lifetime measurements for different interfaces. Lifetime measurements for radiating dipole (RhB) on (a) PC, (B) PC-hBN, (c) PC-AuNP, (d) PC-AuNP-hBN and (e) PC-AuNP-hBN-AuCS. The experiments are performed as per the protocol described in our earlier work using a pulsed 532 nm laser source. A 1 nM concentration of RhB was used for all the lifetime measurements, where the ethanol solution was used as solvent. All the samples were spin coated over the appropriate substrates at 3000 rpm for 60 seconds to mimic the experimental conditions used in this research work and the lifetime measurements were performed under laser 532 nm excitation.

Further, incorporation of the AuCSs on top of the PC+AuNPs+hBN system resulted in the generation of significant reductions in the lifetime (0.64 ns, 40.2%; 1.47 ns, 7.65% and 2.30 ns, 52.1%) indicating multiple channels supporting radiative decay rate, which is seen as experimental enhancement in the overall PC coupled fluorescence observed at the detector [1, 3]. While the cryosoret nano-assemblies act as cavity-like nano-antennas generating hottest hotspots (with 3D field enhancement in space), the out-coupled enhanced emission is steered via the GMR of the underlying PC. These observations of reduction in lifetime with concomitant enhancement in the coupled fluorescence intensity counts are in excellent agreement with observations made in previous reports with the study of different nanomaterials and radiating dipoles over PC substrate

[11, 56-59]. These observations hence point to the multi-modal enhancement strategy created in a single platform where the quenching is mitigated as well as fluorescence is enhanced with high directionality. While the incorporation of plasmonic nanomaterials (as monolayer and cryosoret) support radiating plasmon model, the photonic crystal substrate support radiating GMR model, thereby rendering highly efficient photon management strategy.

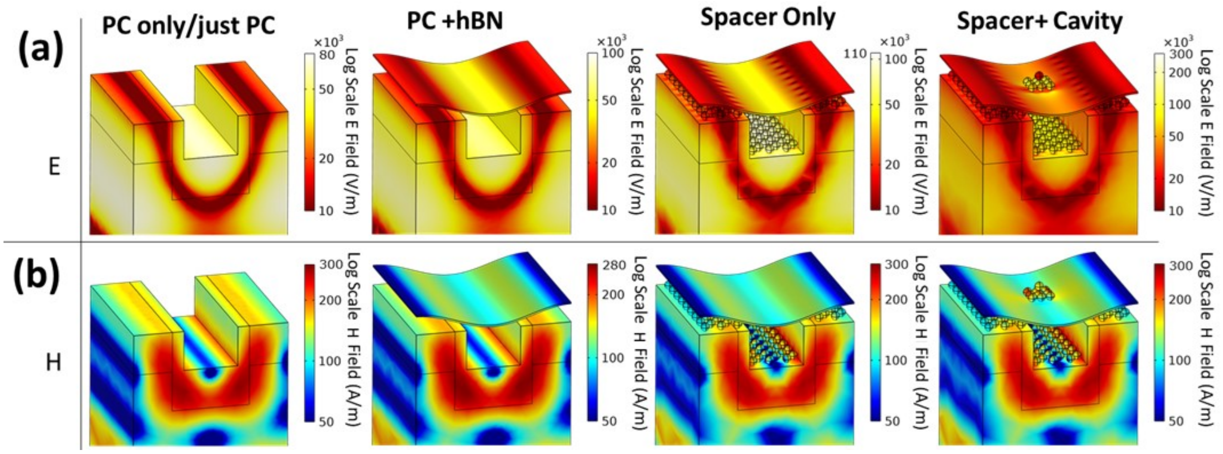

Figure S7. Simulations showing electric and magnetic hotspots. COMSOL Multiphysics simulations showing the (a) electric field distribution and (b) magnetic field distribution of different interfaces, namely PC, PC+hBN, PC+AuNP+hBN, PC+AuNP+hBN + AuCS for TE polarization (TM polarization is presented in Figure 5 of the manuscript).

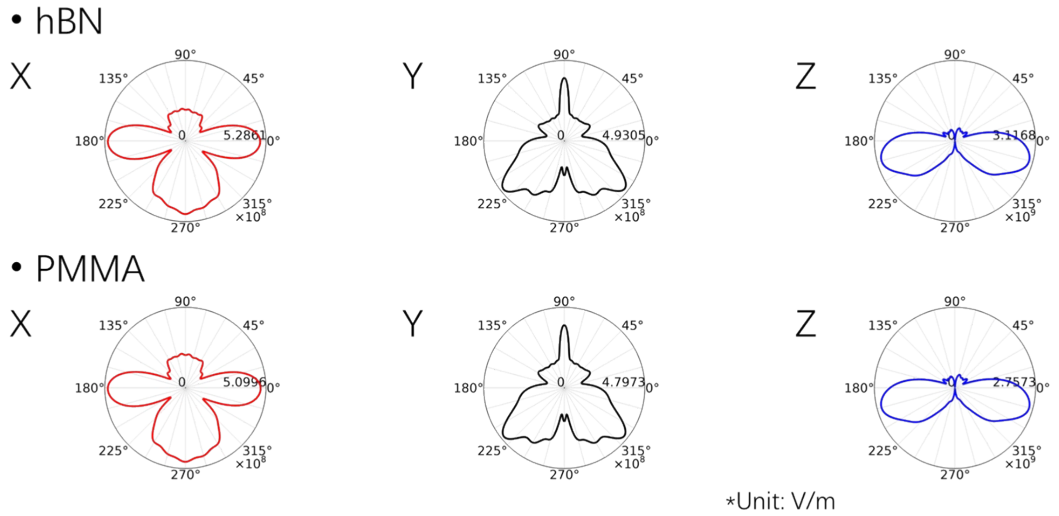

Figure S8. Comparison of dequenching properties of hBN compared to a polymer, PMMA. E-field simulations and respective far-field radiation pattern of for x, y and z-oriented radiating dipole on hBN -PC (top) and PMMA-PC (bottom) interface.

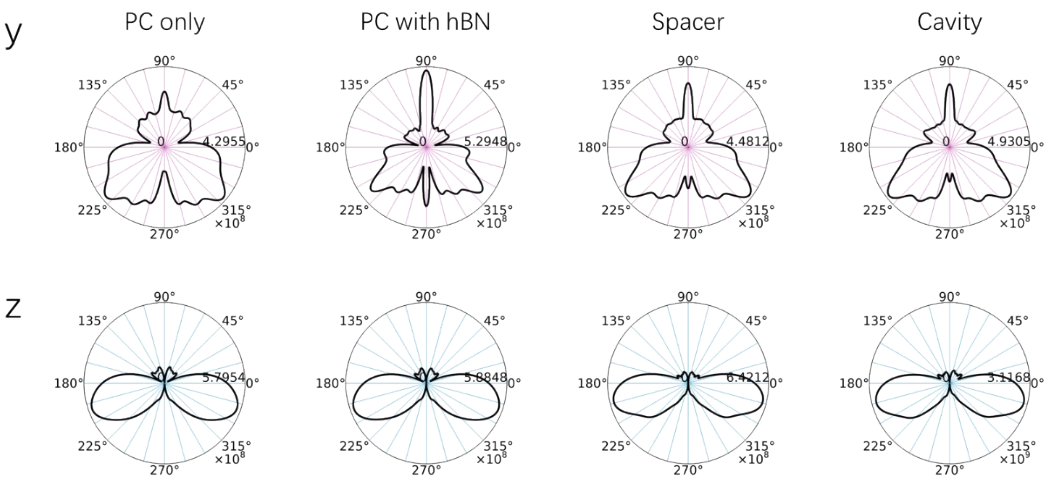

Figure S9. Electric field far-field radiation pattern of the four configurations for y- (top) and z- (bottom) oriented dipoles.

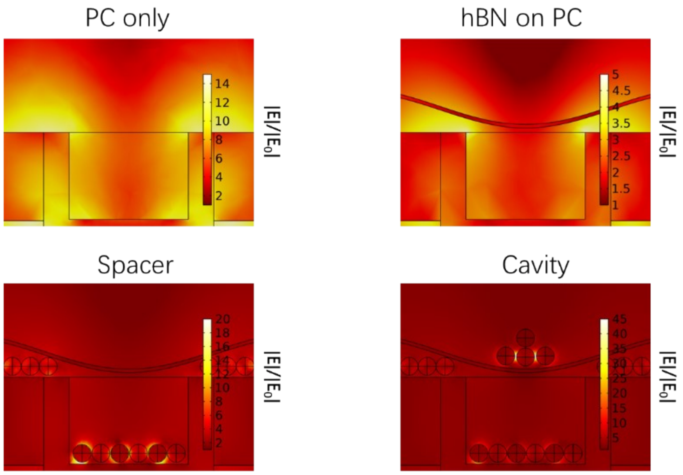

Figure S10 Normalized $|E|/|E_0|$ maps for pure PC, PC with hBN thin film, AuNPs coated PC with hBN thin film, and cavity formed with AuCS on the top of the hBN. Strong near field enhancement is observed at the vicinity of the radiating dipole

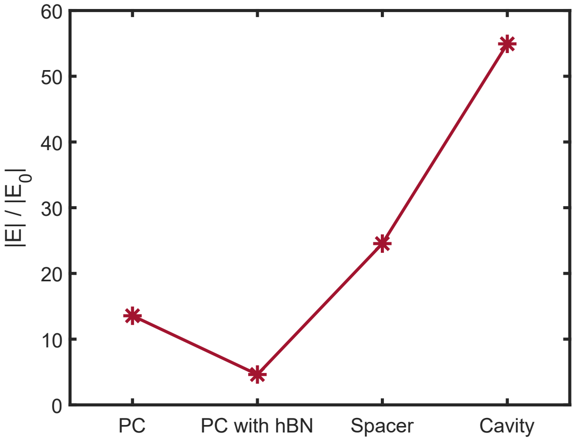

Figure S11. Comparative plot of the maximum enhancement factor in the near field of the simulation for the four cases in Figure S10. The cavity configuration shows the greatest near field enhancement, which is consistent with the emission enhancement.

Future perspectives and outlook

In this research, we demonstrated a unique strategy to mitigate fluorescence quenching which was considered a perennial challenge in the broad domain of plasmon-coupled fluorescence technologies [60-67]. In order to accomplish this, we introduced a versatile and promising nano-architecture with hexagonal boron nitride (hBN) serving as an active spacer layer on top of the AuNP-decorated PC substrate. In addition to avoiding the quenching problem, the hBN layer engendered spatial separation of the metal and the emitter enabling augmentation in fluorescence at optimized distances of PC-plasmonic AuNPs-radiating dipole hybrid systems, hence opening up an entirely new integrated material platform for flatland photonics. Hence, the exploration of different types of 2D materials such as MoS_2, WS_2, and MXenes to name a few is expected to yield interesting optical coupling effects especially when the 2D material itself is fluorescing due to dopant effects [68]. In this perspective, this work charts new research directions where active nano-engineering protocols can be harnessed at PC -2D material interface to understand interesting yet unknown light matter interactions [69-71]. We believe that the current report on hBN -PC research lays the groundwork for construction of multifunctional nano-engineered interfaces with tunable optical properties (Figure S8).

Another interesting outlook emerges from the active research in the generation of library of PC substrates and their optical modes. While we have shown the coupling characteristics of the 1DPC that sustain GMR based modes, the spectrum of PCs that are designed worldwide are very

vast with substrates displaying ultra-high-Q factors, bound states in the continuum (BICs), Rabi splitting, Bloch surface wave, internal optical modes, to name a few with some of them rendering narrower resonances and high spectral selectivity [72-76]. The combination of such effects with low-dimensional materials (such as hBN) is expected synergize the radiating plasmon and GMR model with antenna-coupled effects (generating dark and light modes). We foresee a generation of tunable hybrid modes which would open doors for extremely sensitive biosensing platforms with optimizations of the PC geometries such as lattice symmetry, fill factor and periodicity to synchronize with the optical modes of the low-dimensional materials.

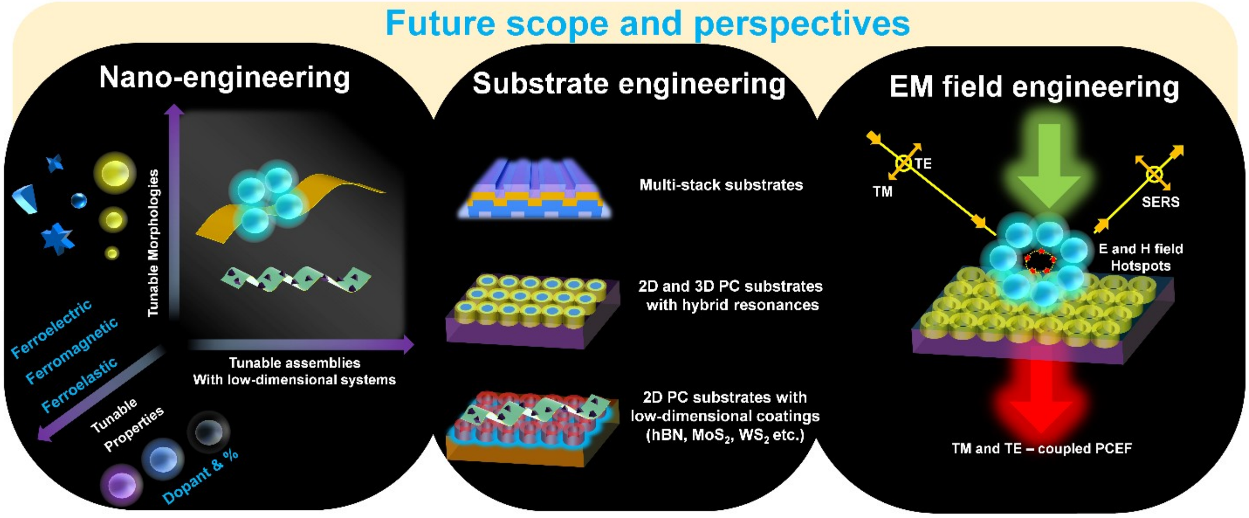

Figure S12. Future scope and perspectives. We envisage three major directions in which this work would be explored in the near and far future. The first direction is related to the different nano-engineering methodologies that can be incorporated to generate hybrid interfaces where 2D materials is expected to perform better when decorated with differently sized and shaped plasmonic and dielectric nanomaterials. The second direction comprises the development of the substrate itself where exploration of other 1DPCs such as Bragg mirrors and 2D and 3D PCs would render interesting photo-plasmonic coupling phenomena emerging from the existing of different types of optical modes in these different substrates. The third direction includes the hybridization of first two approaches of nano-engineering and substrate engineering to develop effective means of light harvesting using plasmonic and dielectric nano-assemblies. The incorporation of nano-assemblies presents options to tap into the full potential of EM radiation where TM and TE coupled modes can be utilized using E-field and H-field hotspots rendered by circulating displacement currents in nano-assemblies. While we have shown a glimpse of possibilities using nano-assemblies considering cryosorets as an example we believe that assemblies with precise nanogap distances would render interesting outcomes that are specifically applicable to detecting molecules in such gaps (tuned to the molecules dimensions) hence enabling ultra-sensitive detection systems that are quench-free.

An exciting direction that our research is expected to take is by modulating the ultrathin wide bandgap hBN material itself where such efforts have already demonstrated their applicability in nanophotonic device fabrication as hBN supports phonon-polariton modes, hyperbolic phonon

polaritons (HPPs) in two distinct Reststrahlen bands [77, 78]. It is important to note that the resonances of PCs can be tuned to UV-vis-NIR regions, and hence the strong field confinement and directional energy propagation effects rendered by hBN in the mid-IR range can be coupled selectively to the modes of the underlying PC substrate. While our architecture has used hBN in the visible region as a robust high refractive index material for dequenching and hybrid plasmonic-PC coupling effects, we believe that embedding defects in hBN would enabling surface emitters due to the generation of single photon sources (SPSs). Its active coupling to PC substrates is expected present room-temperature SPSs with narrow linewidth and ultra-high brightness. Moreover, active doping and embedment of color centers in hBN [79, 80] spacer layers would yield high Purcell enhancement preserving coherence due to the metal-dielectric-metal architecture that can be achieved using PC-AuNP-hBN -AuCS interface. While these aspects are stimulating research directions, it must not go without mention that such efforts would need large optimizations of thicknesses, stacking effects, twisting in multilayers and generation of moiré-induced optical features to name a few.

With the ever-evolving research in the domain of nano-assemblies the explorations with cryosorets and 2D materials still remains a fertile domain. Modifications in the composition of metal-dielectric, meal-magnetic (e.g., Fe_3O_4, CoPt) hybrid cryosorets as well the inter-particle distance, aspect ratio and orientation are expected to yield tunable resonance bandwidth, field entrapment and coupling effects with adjacent fluorescent emitters [81-85]. Magnetically active nano-assemblies when interfaced with carefully engineered PCs is envisioned to enable tuning of coupled fluorescence intensity via magneto-optic Kerr or Faraday effects, especially since the PCs are highly polarization selective. Moreover, coupling of 2D materials like hBN is expected to present stimuli responsive effects when studied using Janus-type cryosorets with chemical and optical anisotropic faces using appropriate linkers and legands, hence opening a new window towards exploring reconfigurable cryosorets for modulating fluorescence (dynamic switching mechanisms for nanophotonic logic or memory elements). Such explorations are expected to be catalyzed beyond the conventional set of molecular emitters such as chemical complexes that have high selectivity to a particular analyte, colloidal quantum dots, nanodiamonds with different types of nitrogen vacancy or silicon-vacancy (SiV) centers, as well as rare-earth doped nanocrystals. Such materials when interfaced with tunable phonon density and optical transparency of hBN (at controlled depths via focused ion beam or laser writing) would render diverse spectral coupling characteristics with longer coherence times compatible for quantum photonics and biosensing.

In summary, we would like to highlight that the integration of top-down nanofabrication for PC patterning and bottom-up assembly for cryosorets presented in this work presents vast opportunities for future research including but not limited to 3D reconfigurability (without lattice mismatch issues) due to van der Waals interfaces, on-chip integration due to PC substrates, integration with microfluidic technologies due to robustness of the platform, electro-optic tuning using gate-controlled hBN/graphene heterostructures enabling voltage-controlled modulation of optical coupling effects. Such experimental research directions are expected to push the computer simulations-based modelling methods such as coupled-mode theory, FDTD, and electromagnetic boundary integral methods to better understand the near-field and far-field coupling between the radiating dipole and hybrid interfaces. While our simulations in this work presents a glimpse towards active PC-plasmonic coupling generating electric and magnetic field hotspots, investigations of phenomena such as slow-light effects (in Bragg mirrors for instance) would demand machine learning-guided optimization of PC grating interfaces, cryosoret hybrids, proximal distance optimizations for radiating dipoles, all of which is expected to lead to intelligent photonic frameworks for next-gen photonic technologies [86]. In conclusion, while this study demonstrates an interesting spacer+cavity hybrid approach for suppressing the quenching effects, the preceding paragraphs presents a blueprint for multifunctional applicability of the current research where interplay of flatland materials, hybrid resonances, and quantum emitters opens a new horizon for nano-optoelectronic technologies.

Table S1. Comparative analysis of the hybrid photonic-plasmonic systems, fluorescence enhancement, analyte detected, and limit of detection from different works with our current research work

Sl. No	Title	Hybrid interface	Fluorescence enhancement	Analyte detected	Limit of detection	Reference
1	Improving upconversion emission of $NaYF_4:Yb^{3+}$, Er^{3+} nanoparticles by coupling Au nanoparticles and photonic crystals: The detection enhancement of Rhodamine B	$NaYF_4:Yb^{3+}$, Er^{3+} NPs deposited on the AuNPs/opal hybrid	35-fold	Rhodamine B	164 µM	[87]
1	Photoluminescence enhancement of carbon dots induced by hybrids of photonic crystals and gold–silver alloy nanoparticles	PMMA opal photonic crystals/Au–Ag alloy plasmon hybrids	53-fold	-	-	[88]
2	Control of plasmonic fluorescence enhancement on self-assembled 2-D colloidal crystals	Ag-capped 2-D colloid crystals	60-fold	-	-	[89]
3	Bloch Surface Waves and Internal Optical Modes-Driven Photonic Crystal-Coupled Emission Platform for	AgNPs on Bragg mirror based Photonic crystal	~200-fold	Al^{3+} ions	1 fM	[38]

	Femtomolar Detection of Aluminum Ions					
4	Boosting fluorescence efficiency via filling technique prepared photonic crystal composites	Au-doped inverse-opal PC (IOPC) with encapsulated Au NPs	242-fold	-	-	90
5	Coupling of Nanoparticle Plasmons with Colloidal Photonic Crystals as a New Strategy to Efficiently Enhance Fluorescence	Coupling of surface plasmons of metal particles with optical properties of colloidal photonic crystals	260-fold	rhodamine B	-	91
6	Label-Free Fluorescence Quantitative Detection Platform on Plasmonic Silica Photonic Crystal Microsphere Array	silica photonic crystal microsphere (SPCM) array with plasmonic AuNPs	370-fold	Aflatoxin B_1 (AFB_1)	0.025 ng/mL	92
7	Purcell Factor: A Tunable Metric for Plasmon-Coupled Fluorescence Emission Enhancements in Cermet Nanocavities	$TiC_{0.5}N_{0.5}$ (TiCN) - Ag hybrid interface	54-fold	Tryptophan	10 nM	93
8	Ag-CNT Architectures for Attomolar Dopamine Detection and 100-Fold Fluorescence Enhancements with Cellphone-Based Surface Plasmon-Coupled Emission Platform	Ag-CNT-Ag hybrid interface	100-fold	Dopamime	1 aM	94
9	Photonic-crystal-enhanced fluorescence: Template-free gold cryosoret nanoassembly steering, dequenching, and augmenting the quenched emission from radiating dipoles	Gold cryosoret - Photonic crystal hybrid interface	200-fold	-	-	25
10	Magnetic “Squashing” of Circulating Tumor Cells on Plasmonic Substrates for Ultrasensitive NIR Fluorescence Detection	Magnetic nanoparticles - plasmonic gold (pGOLD) chip	120-fold	circulating tumor cells (CTCs	≈1 cell mL^{-1}	95
11	DNA-Engineerable Ultraflat-Faceted Core–Shell Nanocuboids with Strong, Quantitative Plasmon-Enhanced Fluorescence Signals for Sensitive, Reliable MicroRNA Detection	Fluorescent silica shell-coated FANCs (FS-FANCs); fluorescence-amplified nanocuboids (FANCs)	~186-fold	miRNA-134; brain-specific biomarker	1.0 fM (40 zmol)	96
12	Femtomolar Detection of Spermidine Using Au Decorated SiO2 Nanohybrid on Plasmon-Coupled Extended Cavity Nanointerface: A Smartphone-Based Fluorescence Dequenching Approach	Au-decorated SiO_2 NPs (AuSil), a metal (Au)-dielectric (SiO_2) hybrid material	207-fold	Spermidine	10 fM	61
13	Photonic crystal band edge coupled enhanced fluorescence from magneto-plasmonic cryosoret nano-assemblies for ultra-sensitive detection	Fe_3O_4–Au cryosoret nano-assemblies	450-fold	Rhodamine B	10 aM	26
15	Current research	hBN (spacer) engineered PC substrate with cryosorets nano-assemblies as hybrid cavity interface	650-fold	Rhodamine B	1 aM	Current work

References:

1. J. R. Lakowicz, K. Ray, M. Chowdhury, H. Szmacinski, Y. Fu, J. Zhang and K. Nowaczyk, *Analyst*, 2008, **133**, 1308-1346.
2. S. Dutta Choudhury, R. Badugu and J. R. Lakowicz, *Acc. Chem. Res.*, 2015, **48**, 2171-2180.
3. J. R. Lakowicz, *Plasmonics*, 2006, **1**, 5-33.
4. J. R. Lakowicz, *Principles of fluorescence spectroscopy*, Springer, 2006.
5. P. K. Badiya, S. G. Patnaik, V. Srinivasan, N. Reddy, C. S. Manohar, R. Vedarajan, N. Mastumi, S. K. Belliraj and S. S. Ramamurthy, *Chemical Physics Letters*, 2017, **685**, 139-145.
6. M. A. Badshah, N. Y. Koh, A. W. Zia, N. Abbas, Z. Zahra and M. W. Saleem, *Nanomaterials*, 2020, **10**, 1749.
7. R. M. Bakker, H.-K. Yuan, Z. Liu, V. P. Drachev, A. V. Kildishev, V. M. Shalaev, R. H. Pedersen, S. Gresillon and A. Boltasseva, *Applied Physics Letters*, 2008, **92**, 043101.
8. M. Bauch, K. Toma, M. Toma, Q. Zhang and J. Dostalek, *Plasmonics*, 2014, **9**, 781-799.
9. E. Fort and S. Grésillon, *J. Phys. D: Appl. Phys.*, 2007, **41**, 013001.
10. S. Bhaskar, *Micromachines*, 2023, **14**, 574.
11. S. Bhaskar, P. Das, V. Srinivasan, S. B. N. Bhaktha and S. S. Ramamurthy, *Materials Research Bulletin*, 2022, **145**, 111558.
12. S. Bhaskar, A. Rai, K. M. Ganesh, R. Reddy, N. Reddy and S. S. Ramamurthy, *Langmuir*, 2022, **38**, 12035-12049.
13. S.-H. Cao, W.-P. Cai, Q. Liu, K.-X. Xie, Y.-H. Weng, S.-X. Huo, Z.-Q. Tian and Y.-Q. Li, *Journal of the American Chemical Society*, 2014, **136**, 6802-6805.
14. S.-H. Cao, Y.-H. Weng, K.-X. Xie, Z.-C. Wang, X.-H. Pan, M. Chen, Y.-Y. Zhai, L.-T. Xu and Y.-Q. Li, *ACS Applied Bio Materials*, 2019, **2**, 625-629.
15. S. Bhaskar, P. Das, M. Moronshing, A. Rai, C. Subramaniam, S. B. N. Bhaktha and S. S. Ramamurthy, *Nanophotonics*, 2021, **10**, 3417-3431.
16. J. R. Lakowicz, *Analytical Biochemistry*, 2005, **337**, 171-194.
17. S. Bhaskar, N. C. S. S. Kowshik, S. P. Chandran and S. S. Ramamurthy, *Langmuir*, 2020, **36**, 2865-2876.
18. S.-H. Cao, W.-P. Cai, Q. Liu and Y.-Q. Li, *Annual Review of Analytical Chemistry*, 2012, **5**, 317-336.
19. M. H. Chowdhury, K. Ray, C. D. Geddes and J. R. Lakowicz, *Chemical Physics Letters*, 2008, **452**, 162-167.
20. K. M. Ganesh, S. Bhaskar, V. S. K. Cheerala, P. Battampara, R. Reddy, S. C. Neelakantan, N. Reddy and S. S. Ramamurthy, *Nanomaterials*, 2024, **14**, 111.
21. Z. Gryczynski, I. Gryczynski, E. Matveeva, J. Malicka, K. Nowaczyk and J. R. Lakowicz, in *Methods in Cell Biology*, Academic Press, 2004, vol. 75, pp. 73-104.
22. Q. Liu, S.-H. Cao, W.-P. Cai, X.-Q. Liu, Y.-H. Weng, K.-X. Xie, S.-X. Huo and Y.-Q. Li, *The Journal of Physical Chemistry B*, 2015, **119**, 2921-2927.
23. R. Badugu, J. Mao, D. Zhang, E. Descrovi and J. R. Lakowicz, *J. Phys. Chem. C*, 2020, **124**, 22743-22752.
24. S. Bhaskar, S. M. Lis S, S. Kanvah, S. Bhaktha B. N and S. S. Ramamurthy, *ACS Appl. Opt. Mater.*, 2023, **1**, 159-172.
25. S. Bhaskar, L. Liu, W. Liu, J. Tibbs and B. T. Cunningham, *MRS Bull.*, 2025, DOI: 10.1557/s43577-024-00850-2.

26. S. Bhaskar, L. Liu, W. Liu, J. Tibbs, L. D. Akin, A. Bacon and B. T. Cunningham, *APL Materials*, 2025, **13**.
27. S. Bhaskar, W. Liu, J. Tibbs and B. T. Cunningham, *Applied Physics Letters*, 2024, **124**, 161102.
28. P. Barya, Y. Xiong, S. Shepherd, R. Gupta, L. D. Akin, J. Tibbs, H. Lee, S. Singamaneni and B. T. Cunningham, *Small*, 2023, **19**, 2207239.
29. I. D. Block, P. C. Mathias, N. Ganesh, S. I. Jones, B. R. Dorvel, V. Chaudhery, L. O. Vodkin, R. Bashir and B. T. Cunningham, *Optics Express*, 2009, **17**, 13222-13235.
30. V. Chaudhery, S. George, M. Lu, A. Pokhriyal and B. T. Cunningham, *Sensors*, 2013, **13**, 5561-5584.
31. W. Chen, K. D. Long, H. Yu, Y. Tan, J. S. Choi, B. A. Harley and B. T. Cunningham, *Analyst*, 2014, **139**, 5954-5963.
32. B. T. Cunningham, Frontiers in Pathogen Detection: From Nanosensors to Systems, 2010.
33. N. Ganesh, I. D. Block, P. C. Mathias, W. Zhang, E. Chow, V. Malyarchuk and B. T. Cunningham, *Optics Express*, 2008, **16**, 21626-21640.
34. N. Ganesh, W. Zhang, P. C. Mathias, E. Chow, J. a. N. T. Soares, V. Malyarchuk, A. D. Smith and B. T. Cunningham, *Nature Nanotechnology*, 2007, **2**, 515-520.
35. G. Barbillon, *Nanoplasmonics: Fundamentals and Applications*, BoD – Books on Demand, 2017.
36. J. A. Fan, C. Wu, K. Bao, J. Bao, R. Bardhan, N. J. Halas, V. N. Manoharan, P. Nordlander, G. Shvets and F. Capasso, *Science*, 2010, **328**, 1135-1138.
37. C. D. Geddes, *Surface plasmon enhanced, coupled and controlled fluorescence*, John Wiley & Sons, 2017.
38. S. Bhaskar, P. Das, V. Srinivasan, S. Bhaktha B. N and S. S. Ramamurthy, *J. Phys. Chem. C*, 2020, **124**, 7341-7352.
39. S. Bhaskar, N. S. Visweswar Kambhampati, K. M. Ganesh, M. S. P, V. Srinivasan and S. S. Ramamurthy, *ACS Applied Materials & Interfaces*, 2021, **13**, 17046-17061.
40. C. D. Geddes, *Surface Plasmon Enhanced, Coupled and Controlled Fluorescence*, John Wiley & Sons, 2017.
41. T. Ming, H. Chen, R. Jiang, Q. Li and J. Wang, *J. Phys. Chem. Lett.*, 2012, **3**, 191-202.
42. S.-Y. Lee, T.-Y. Jeong, S. Jung and K.-J. Yee, *Physica Status Solidi (b)*, 2019, **256**, 1800417.
43. V. S. K. Cheerala, K. M. Ganesh, S. Bhaskar, S. S. Ramamurthy and S. C. Neelakantan, *Langmuir*, 2023, DOI: 10.1021/acs.langmuir.3c00801.
44. L. Liu, S. Bhaskar and B. T. Cunningham, *Applied Physics Letters*, 2024, **124**, 234101.
45. A. Rai, S. Bhaskar, K. M. Ganesh and S. S. Ramamurthy, *ACS Applied Nano Materials*, 2022, **5**, 12245-12264.
46. A. Bek, R. Jansen, M. Ringler, S. Mayilo, T. A. Klar and J. Feldmann, *Nano Letters*, 2008, **8**, 485-490.
47. S. Bhaskar, P. Jha, C. Subramaniam and S. S. Ramamurthy, *Physica E: Low-dimensional Systems and Nanostructures*, 2021, **132**, 114764.
48. Y. Li, H. Li, H. Zheng, H. Wu, K. Liu, J. Wang, C. Yang, X. Ma and C. Sun, *Sensors and Actuators B: Chemical*, 2022, **364**, 131880.
49. K.-X. Xie, Q. Liu, S.-S. Jia and X.-X. Xiao, *Analytica Chimica Acta*, 2021, **1144**, 96-101.
50. K.-X. Xie, Q. Liu, X.-L. Song, R.-P. Huo, X.-H. Shi and Q.-L. Liu, *Analytical Chemistry*, 2021, **93**, 3671-3676.

51. S. Bhaskar, Y. Xiong, S. Shepherd, J. Tibbs, A. K. Bacon, W. Liu, L. D. Akin, T. Ayupova, W. Wang, H. Lee, L. Liu, A. Tan, K. Khemtonglang, X. Wang and B. T. Cunningham, in *Nano-Engineering at Functional Interfaces for Multi-Disciplinary Applications*, Elsevier, 2025, pp. 123-156.
52. S. Bhaskar, R. Patra, N. C. S. S. Kowshik, K. M. Ganesh, V. Srinivasan, P. Chandran S and S. S. Ramamurthy, *Physica E: Low-dimensional Systems and Nanostructures*, 2020, **124**, 114276.
53. A. Rai, S. Bhaskar, K. M. Ganesh and S. S. Ramamurthy, *Materials Chemistry and Physics*, 2022, **285**, 126129.
54. Q. Cai, S. Mateti, K. Watanabe, T. Taniguchi, S. Huang, Y. Chen and L. H. Li, *ACS Applied Materials & Interfaces*, 2016, **8**, 15630-15636.
55. G. Kim, M. Kim, C. Hyun, S. Hong, K. Y. Ma, H. S. Shin and H. Lim, *ACS Nano*, 2016, **10**, 11156-11162.
56. S. Bhaskar, V. Srinivasan and S. S. Ramamurthy, *ACS Applied Nano Materials*, 2023, **6**, 1129-1145.
57. S. Bhaskar, D. Thacharakkal, S. S. Ramamurthy and C. Subramaniam, *ACS Sustainable Chemistry & Engineering*, 2023, **11**, 78-91.
58. D. Thacharakkal, S. Bhaskar, T. Sharma, G. Rajaraman, S. Sathish Ramamurthy and C. Subramaniam, *Chemical Engineering Journal*, 2023, DOI: 10.1016/j.cej.2023.148166, 148166.
59. S. M. L. S, S. Bhaskar, R. Dahiwadkar, S. Kanvah, S. S. Ramamurthy and S. Bhaktha B. N, *ACS Applied Nano Materials*, 2023, **6**, 19312-19326.
60. P. Anger, P. Bharadwaj and L. Novotny, *Phys. Rev. Lett.*, 2006, **96**, 113002.
61. S. Bhaskar, N. C. S. S. Kowshik, S. P. Chandran and S. S. Ramamurthy, *Langmuir*, 2020, **36**, 2865-2876.
62. L. Brus, *Phys. Rev. B*, 1996, **53**, 4649-4656.
63. G. Damiano, B. Sara, J. Riccardo, M. Marco, P. Luca, R. Enrico and Z. Nelsi, *Angewandte Chemie*, 2013, **52**, 5965-5968.
64. E. Dulkeith, A. C. Morteani, T. Niedereichholz, T. A. Klar, J. Feldmann, S. A. Levi, F. C. J. M. van Veggel, D. N. Reinhoudt, M. Möller and D. I. Gittins, *Phys. Rev. Lett.*, 2002, **89**, 203002.
65. R. Faggiani, J. Yang and P. Lalanne, *ACS Photonics*, 2015, **2**, 1739-1744.
66. N. Ganesh, P. C. Mathias, W. Zhang and B. T. Cunningham, *Journal of Applied Physics*, 2008, **103**, 083104.
67. N. Kongsuwan, A. Demetriadou, R. Chikkaraddy, F. Benz, V. A. Turek, U. F. Keyser, J. J. Baumberg and O. Hess, *ACS Photonics*, 2018, **5**, 186-191.
68. S. Bhattacharya, J. Boyd, S. Reichardt, V. Allard, A. H. Talebi, N. Maccaferri, O. Shenderova, A. L. Lereu, L. Wirtz, G. Strangi and R. M. Sankaran, *Nature Communications*, 2025, **16**, 444.
69. *Nature Electronics*, 2021, **4**, 699-699.
70. Y. Li, Z. Li, C. Chi, H. Shan, L. Zheng and Z. Fang, *Advanced Science*, 2017, **4**, 1600430.
71. M. Ahmed, S. Moustafa, N. M. Shaalan, M. Rashad and H. Fares, *Colloids and Surfaces A: Physicochemical and Engineering Aspects*, 2025, **724**, 137391.
72. S. Bhaskar, W. Wang, H. Lee, L. Liu, S. Umrao, W. Liu, A. Bacon, J. Tibbs, K. Khemtonglang, A. Tan, T. Ayupova, W.-C. Chen, X. Wang and B. T. Cunningham, *Chemical Reviews*, 2025, DOI: 10.1021/acs.chemrev.4c00653.

73. V. I. Klimov, *Nanocrystal Quantum Dots*, CRC Press, 2017.
74. C. Lopez, *Advanced Materials*, 2003, **15**, 1679-1704.
75. S. Noda and T. Baba, *Roadmap on Photonic Crystals*, Springer Science & Business Media, 2003.
76. H. E. Ruda and N. Matsuura, in *Optical Properties of Materials and Their Applications*, John Wiley & Sons, Ltd, 2019, pp. 251-268.
77. W. Hutchins, S. Zare, D. M. Hirt, J. A. Tomko, J. R. Matson, K. Diaz-Granados, M. Long, M. He, T. Pfeifer, J. Li, J. H. Edgar, J.-P. Maria, J. D. Caldwell and P. E. Hopkins, *Nature Materials*, 2025, **24**, 698-706.
78. Q. Guo, I. Esin, C. Li, C. Chen, G. Han, S. Liu, J. H. Edgar, S. Zhou, E. Demler, G. Refael and F. Xia, *Nature*, 2025, **639**, 915-921.
79. S. S. Mohajerani, S. Chen, A. Alaei, T. Chou, N. Liu, Y. Ma, L. Xiao, S. S. Lee, E.-H. Yang and S. Strauf, *ACS Photonics*, 2024, **11**, 2359-2367.
80. F. Ren, Z. Xu, Y. Wu, X. Wang, Y. Guo, L. Chen, Y. Song and B. Dong, *ACS Applied Nano Materials*, 2024, **7**, 3436-3444.
81. S. R. Ahmed, É. Nagy and S. Neethirajan, *RSC Advances*, 2017, **7**, 40849-40857.
82. C. Alexander, *Faraday Discuss.*, 2013, **166**, 449.
83. K. J. M. Bishop, C. E. Wilmer, S. Soh and B. A. Grzybowski, *Small*, 2009, **5**, 1600-1630.
84. M. A. Boles, M. Engel and D. V. Talapin, *Chemical Reviews*, 2016, **116**, 11220-11289.
85. T.-H. Chen and W.-L. Tseng, *Analytical Chemistry*, 2017, **89**, 11348-11356.
86. *Nano-Engineering at Functional Interfaces for Multi-Disciplinary Applications*, Elsevier, 2025.
87. Y. Ma, J. Zhu, Z. Yang, H. Zhang, J. Qiu and Z. Song, *Journal of Alloys and Compounds*, 2019, **788**, 1265-1273.
88. H. Wang, X. Sun, T. Zhang, X. Chen, J. Zhu, W. Xu, X. Bai, B. Dong, H. Cui and H. Song, *J. Mater. Chem. C*, 2018, **6**, 147-152.
89. W. Hong, Y. Zhang, L. Gan, X. Chen and M. Zhang, *J. Mater. Chem. C*, 2015, **3**, 6185-6191.
90. Y. Qin, X. Zhao, Y. Wang, J. He, Z. Zhu, T. Zhao and G. Dong, *Scientific Reports*, 2025, **15**, 19990.
91. C.-a. Tao, W. Zhu, Q. An, H. Yang, W. Li, C. Lin, F. Yang and G. Li, *J. Phys. Chem. C*, 2011, **115**, 20053-20060.
92. S. Dai, W. Li, R. Xu, X. Wang, Q. Li, M. Dou and J. Li, *Analytical Chemistry*, 2022, **94**, 17939-17946.
93. V. Srinivasan and S. S. Ramamurthy, *J. Phys. Chem. C*, 2016, **120**, 2908-2913.
94. P. K. Badiya, V. Srinivasan, T. P. Jayakumar and S. S. Ramamurthy, *ChemPhysChem*, 2016, **17**, 2791-2794.
95. R. Zhang, B. Le, W. Xu, K. Guo, X. Sun, H. Su, L. Huang, J. Huang, T. Shen, T. Liao, Y. Liang, J. X. J. Zhang, H. Dai and K. Qian, *Small Methods*, 2019, **3**, 1800474.
96. J.-H. Hwang, S. Park, J. Son, J. W. Park and J.-M. Nam, *Nano Letters*, 2021, **21**, 2132-2140.
